# Hydrogen Evolution Electrocatalysis with a Molecular Cobalt Bis(alkylimidazole)methane Complex in DMF: a Critical Activity Analysis

**DOI:** 10.1002/cssc.202201308

**Published:** 2022-10-26

**Authors:** Sander D. de Vos, Maartje Otten, Tim Wissink, Daniël L. J. Broere, Emiel J. M. Hensen, Robertus J. M. Klein Gebbink

**Affiliations:** ^1^ Organic Chemistry and Catalysis Institute for Sustainable and Circular Chemistry Utrecht University Universiteitsweg 99 3584 CG Utrecht (The Netherlands; ^2^ Chemical Engineering and Chemistry Laboratory of Inorganic Materials and Catalysis Department of Chemical Engineering and Chemistry Eindhoven University of Technology P.O. Box 513 5600 MB Eindhoven (The Netherlands

**Keywords:** Cobalt, electrocatalysis, electrode materials, electrolysis, hydrogen evolution

## Abstract

[Co(HBMIM^Ph2^)_2_](BF_4_)_2_ (**1**) [HBMIM^Ph2^=bis(1‐methyl‐4,5‐diphenyl‐1*H*‐imidazol‐2‐yl)methane] was investigated for its electrocatalytic hydrogen evolution performance in DMF using voltammetry and during controlled potential/current electrolysis (CPE/CCE) in a novel in‐line product detection setup. Performances were benchmarked against three reported molecular cobalt hydrogen evolution reaction (HER) electrocatalysts, [Co(dmgBF_2_)_2_(solv)_2_] (**2**) (dmgBF_2_=difluoroboryldimethylglyoximato), [Co(TPP)] (**3**) (TPP=5,10,15,20‐tetraphenylporphyrinato), and [Co(bapbpy)Cl](Cl) (**4**) [bapbpy=6,6′‐bis‐(2‐aminopyridyl)‐2,2′‐bipyridine], showing distinct performances differences with **1** being the runner up in H_2_ evolution during CPE and the best catalyst in terms of overpotential and Faradaic efficiency during CCE. After bulk electrolysis, for all of the complexes, a deposit on the glassy carbon electrode was observed, and post‐electrolysis X‐ray photoelectron spectroscopy (XPS) analysis of the deposit formed from **1** demonstrated only a minor cobalt contribution (0.23 %), mainly consisting of Co^2+^. Rinse tests on the deposits derived from **1** and **2** showed that the initially observed distinct activity was (partly) preserved for the deposits. These observations indicate that the molecular design of the complexes dictates the features of the formed deposit and therewith the observed activity.

## Graphical abstract

Put down a deposit: The electrocatalytic hydrogen evolution performance of molecular complex [Co(HBMIM^Ph2^)_2_](BF_4_)_2_ (**1**) is studied in DMF. Bulk electrolysis demonstrates a competitive performance of **1** towards reported electrocatalysts in terms of H_2_ production and overpotential. Post‐electrolysis analysis reveals that a formed electrode deposit with minor cobalt content also significantly contributes to the observed hydrogen evolution activity.

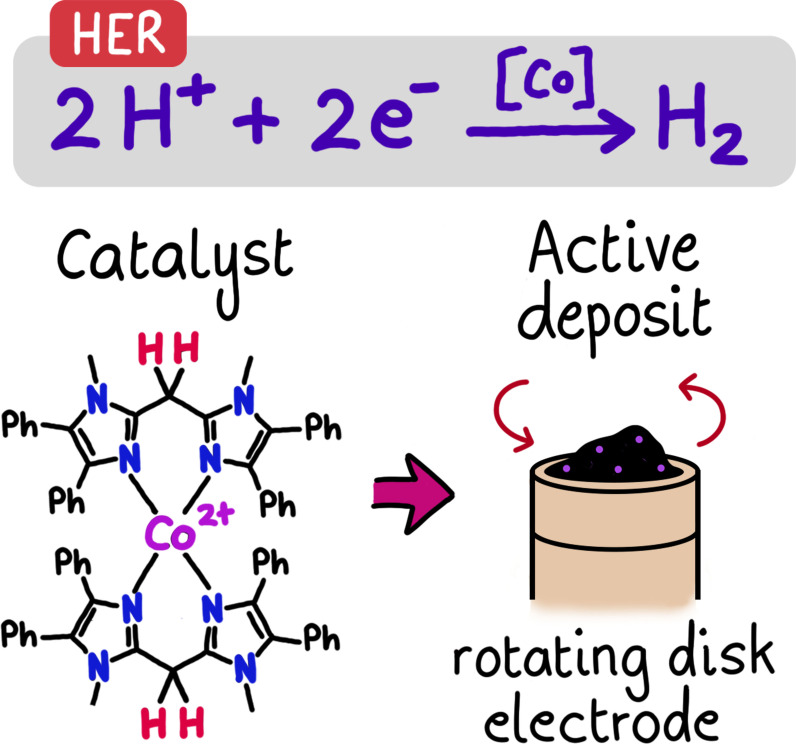

## Introduction

Hydrogen generation from carbon‐neutral sources is an important part of a multifaceted strategy to meet growing global energy demands.^[1]^ A promising approach is the electrocatalytic splitting of water into oxygen and protons and follow‐up hydrogen formation.^[2]^ To facilitate the hydrogen evolution reaction (HER), Pt is the catalytic material of choice because of its high activity and low overpotential.^[3]^ However there remains a strong interest to move to cheaper and more abundant catalyst materials.^[4]^ Approaches include the use of carbon‐based electrode materials with deposited first‐row transition metals,^[5]^ and the use of coordination complexes of first‐row transition metals able to catalyze the HER at a potential close to the standard potential of the H^+^/H_2_ couple.^[6]^ Coordination complexes allow for precise molecular designs, leading to enhanced selectivity and activity as well as mechanistic insights on key intermediates.^[7]^ In particular, cobalt‐based complexes gained significant attention because of their HER activity at low overpotentials.^[8]^ The electrocatalytic performance of molecular complexes is typically assessed using a combination of voltammetry and bulk electrolysis in organic solvents or water using a sacrificial proton donor. One disadvantage is the fragility of molecular complexes under the electro‐reductive conditions, sometimes changing the initial molecular complex into a structurally distinct active catalyst.^[9]^ Over the last decade, the molecular electrocatalysis field, encouraged by the work of Savéant and co‐workers, developed an increased understanding of the voltammetric responses and the identification of the true electrocatalytic species.^[10]^ Complementary to voltammetry, bulk electrolysis allows for the generation of sufficient hydrogen to determine catalytic rates and Faradaic efficiencies. However, monitoring the chemical integrity of the catalyst during electrolysis and identifying the chemical structure of the active species in catalysis remains challenging.[^9b^, ^11^] A number of studies have focused on transformations of molecular complexes during HER catalysis and reveal that catalyst as well as electrode modifications can take place to form active materials with distinct chemical compositions.[^11b^, ^12^]

**Figure 1:**
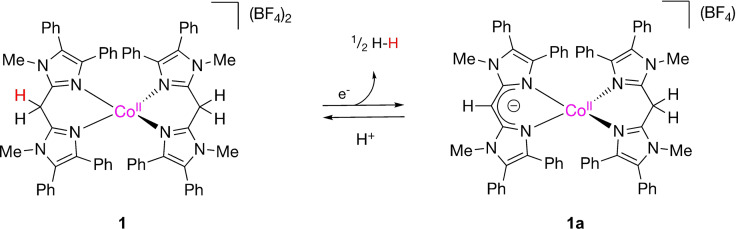
Proposed ligand‐mediated HER reactivity for **1**.

Recently, our group has reported on the dicationic cobalt complex [Co(HBMIM^Ph2^)_2_](BF_4_)_2_ (**1**), comprising two neutral HBMIM^Ph2^ diimine ligands, and its use as a HER electrocatalyst.^[13]^ Cyclic voltammetry experiments showed an irreversible, peak‐shaped reductive response for **1** in MeCN at *E*_p_=−1.96 V vs. Fc^+^/Fc, which was assigned to the fast reduction of Co^II^ to Co^I^. Combined experimental and density functional theory (DFT) studies supported that **1** undergoes H‐atom loss upon reduction (either electrochemically or chemically using KC_8_) and consecutive H_2_ formation, resulting in formal deprotonation and generation of cobalt complex [Co(HBMIM^Ph2^)(BMIM^Ph2–^)](BF_4_) (**1 a**), containing one deprotonated BMIM^Ph2−^ ligand (Figure 1). Under catalytic conditions, protonation restores complex **1** and closes a catalytic cycle. Isolation and characterization of **1 a** in combination with mechanistic investigations using DFT suggested that dihydrogen formation proceeds via the intramolecular combination of an intermediate Co^III^−H moiety with a ligand C−H proton, providing a unique HER mechanism. Robustness tests were carried out by means of bulk electrolysis with acetic acid (as proton source) in MeCN using a mercury electrode and demonstrated that **1** suffered from fast deactivation with a total turnover number of 0.25. Nevertheless, the involvement of the ligand C−H moiety was further demonstrated because a dimethylated analogue of **1**, devoid of a proton relay functionality in the ligand backbone, is not catalytically active.

Here, we report on a detailed study of the electrochemical behavior of complex **1** and its use in electrocatalytic hydrogen production during electrolysis studies in both MeCN and DMF solution. We particularly sought to optimize the electrocatalytic conditions using analytical electrochemical sweeping voltammetry techniques.^[14]^ Subsequent bulk electrolysis experiments were performed in a newly designed electrocatalytic HER‐model cell to allow for precise monitoring of the current density, quantitative H_2_ product formation via fast inline gas chromatography (GC) analysis, and catalyst stability over time. The performance of **1** was then benchmarked to three established molecular catalysts in the field. Overall, this study provides comparative and quantitative insight in the electrocatalytic performance of molecular cobalt complexes in non‐aqueous HER and sheds light on the stability of the complexes in a bulk‐electrolysis set‐up.

## Results and Discussion

### Electrochemical properties

**Figure 2:**
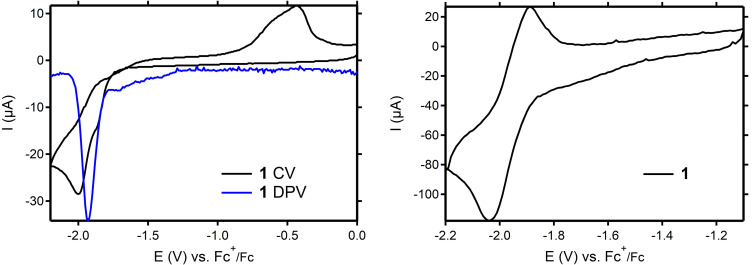
Left: CV (black) and DPV (blue) of **1** (2 mm) in DMF (containing 0.1 m *n*Bu_4_NBF_4_ as supporting electrolyte) at 100 mV s^−1^. Right: CV of **1** (2 mM) in DMF (0.1 m *n*Bu_4_NBF_4_) at 2000 mV s^−1^. Potentials in V vs. Fc^+^/Fc; working electrode: glassy carbon; counter‐electrode: Pt wire; reference electrode: Ag/Ag(NO_3_).

Following our initial investigation in MeCN solution,^[13]^ we have investigated the electrochemical properties of **1** in DMF solution. The cyclic voltammogram (CV) and differential pulse voltammogram (DPV) of **1**, recorded at a glassy carbon (GC) electrode in DMF (0.1 m *n*Bu_4_NBF_4_) at 100 mV s^−1^, display one irreversible reductive response at peak potentials of *E*_p_=−2.00 V and *E*_p_=−1.93 V vs. Fc^+^/Fc, respectively (Figure 2, left). The reductive response was assigned to the Co^II^/Co^I^ redox couple coupled with a follow‐up chemical reaction, presumably a formal loss of a methylene H‐atom (overall deprotonation), in line with our earlier studies on **1** in MeCN (see above).^[13]^ Increasing the scan rate in CV up to 2000 mV s^−1^, the irreversible nature of the reductive response changes to a well‐defined and quasi‐reversible redox couple at a half‐wave potential *E*_1/2_ of −1.96 V vs. Fc^+^/Fc (Δ*E*_p_=130 mV) (Figure 2, right). This observation indicates that the electrochemical oxidation of reduced **1** becomes feasible at higher scan rates in DMF, suggesting that the chemical reactivity is impaired on this timescale. Surprisingly, this behavior is not consistent between solvents, as parallel experiments in MeCN do not show any quasi‐reversible responses up to scan rates of 2000 mV s^−1^, corroborating the increased stability of reduced **1** in DMF (Figure S1).

Next, we sought to obtain further quantitative data on the electrochemical behavior of **1** in DMF. Using the Randles–Sevcik equation, the diffusion coefficient of **1** was determined through a variable scan rate study (Figure S2), affording a value of 3.5×10^−6^ cm^2^ s^−1^ (see the Supporting Information), which is in the same order of magnitude as related molecular cobalt(II) complexes ranging from 3.4 to 8.2×10^−6^ cm^2^ s^−1^.[^8f^, ^15^] When plotting the peak currents *i*_p_ vs. the square root of the scan rate, a good linear fit is observed (Figure S3), which indicates that electron transfer between the electrode and **1** takes place in a truly homogeneous fashion.^[16]^ Plots of the anodic and cathodic peak potentials (*E*_p,a_ and *E*_p,c_) versus the logarithm of the scan rate ranging from 2000 to 20000 mV s^−1^ show linear trends to more anodic/cathodic potentials, indicating a slow electron transfer rate (Figure S3).^[16]^ Unbalanced slopes of opposite signs (+40 and −130 mV dec^−1^) were determined for the Co^II^/Co^I^ couple, highlighting an asymmetry of the energy barrier for electron transfer.^[10]^ These observations lend further credit to the relative ease with which **1** can be reduced and undergo a subsequent chemical transformation, while the reversed oxidation reaction is relatively slow. Since this behavior is well suited for electrocatalysis, we continued our investigations by examining the electrocatalytic HER properties.

### Electrocatalytic properties

**Figure 3:**
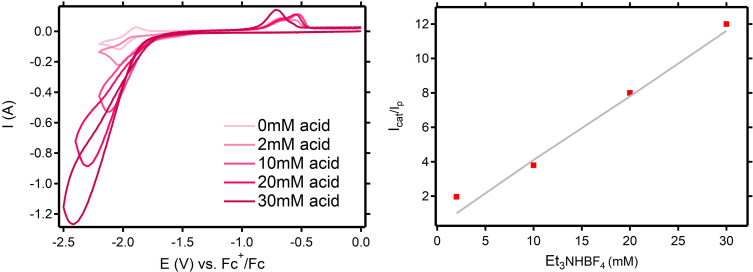
Left: CV of **1** (2 mm) recorded in the presence of increasing amounts of Et_3_NHBF_4_: 2, 5, 10, 20, 30 mm in DMF (0.1 m *n*Bu_4_NBF_4_). Potentials in V vs. Fc^+^/Fc. Conditions: scan rate 2000 mV s^−1^; working electrode: glassy carbon; counter‐electrode: Pt wire; reference electrode: Ag/Ag(NO_3_). Right: corresponding plot of *i*_c_/*i*_p_ vs. Et_3_NHBF_4_ concentration.

The electrocatalytic activity of **1** towards hydrogen evolution was initially examined by recording CVs in DMF using *n*Bu_4_NBF_4_ as supporting electrolyte and increasing amounts of a sacrificial proton source. Two proton sources with distinct acid dissociation constants and standard potentials for the HA/A^−^,H_2_ half reaction in DMF were selected: triethylammonium Et_3_NHBF_4_ (p*K*_a_=9.2, *E*°_HA_=−1.31 V) and phenol (p*K*_a_=>18, *E*°_HA_=<−1.83 V).^[17]^ First, the stability of **1** in the presence of increasing amounts of the strongest acid (Et_3_NHBF_4_) was monitored by ^1^H nuclear magnetic resonance (NMR) spectroscopy, showing no signs of protonation nor degradation (Figures S4 and S5). Therefore, it is expected that **1** needs to be reduced first before participating in a chemical reaction involving a proton. Figure 3 shows the CVs of **1** in the presence of increasing amounts of Et_3_NHBF_4_. The addition of acid triggers the development of a “peak‐shaped” catalytic wave at the Co^II^/Co^I^ couple, regardless of acid concentration and with complete loss of the coupled oxidative response. The catalytic peak current (*i*_cat_) is proportional to the acid concentration (Figure S6), and the catalytic wave keeps increasing over the whole range of acid concentrations (2–30 mm), without reaching an upper limit. The Co^II^/Co^I^ reductive response (*E*_p,c_) did not progressively shift towards more anodic potentials, which indicates that the complex needs to be reduced first before participating in a subsequent chemical reaction such as protonation.^[18]^ This observation agrees with the unaltered ^1^H‐NMR spectra of **1** in the presence of excess Et_3_NHBF_4_. In the presence of Et_3_NHBF_4_ (5 equiv.) a scan rate study was performed, in which the peak current (*i*_cat_) remained proportional to the square root of the scan rate (Figure S7). This observation indicates that the catalytic process is diffusion limited under the applied conditions.^[10]^ Moreover, during bulk electrolysis experiments it became evident that the direct acid reduction by the bare glassy carbon electrode hardly contributes to the overall HER activity (see below).

A plot of the ratio between the catalytic currents over the initial complex current (*i*_cat_/*i*_p_) and the acid concentration yields a linear correlation (Figure 3, right), from which the *k*_obs_ (turnover frequency, TOF) was determined using Equation 1.^[19]^

(1) $$k_{obs}\left(TOF\right)=1.94\times v\times {\left(\frac{i_{cat}}{i_{p}}\right)}^{2}$$

In the presence of 2, 10, 20, and 30 mm Et_3_NHBF_4_ (*E*°_HA_=−1.31 V),^[17]^ the catalytic current corresponds to a *k*_obs_ (TOF) of 15, 57, 269, and 555 s^−1^, respectively. The catalysis experiment was also carried out with the much weaker proton source phenol (*E*°_HA_=<−1.83 V)^[17]^ in DMF (Figure S8), demonstrating a lower catalytic rate at a lower overpotential (*k*_obs_ (TOF) dropped almost 10‐fold), which is consistent with the scaling relationship between rate and overpotential in electrocatalysis and confirms that **1** reacts as a true electrocatalyst.^[20]^ The absolute catalytic rate for **1** in the presence of 30 mm Et_3_NHBF_4_ in DMF on the CV time scale (TOF=555 s^−1^) is an increase as compared to the previously reported HER performance of **1** in MeCN (160 equiv. AcOH (*E*°_HA_=<−1.46 V), TOF=200 s^−1^).^[13]^ The CV of the acid alone shows a weak electrocatalytic response at more negative potentials (Figure S9).

### Bulk electrolysis

Next, bulk electrolysis experiments were performed in a custom‐made two‐compartment HER model‐cell (Figure S10), using a glassy carbon rotating‐disk electrode (RDE) with a well‐defined surface area (0.196 cm^2^) as working electrode (cathode) operating at 0 or 2000 rpm. In‐situ H_2_ quantification was achieved by in‐line measurements using a GC thermal conductivity detector (TCD) apparatus (see the Supporting Information). The electrolysis cell design has been reported for solid‐state electrocatalyst and we have adopted the design to be able to assess the electrocatalytic HER performance for molecular complexes in solution.^[5]^

**Figure 4:**
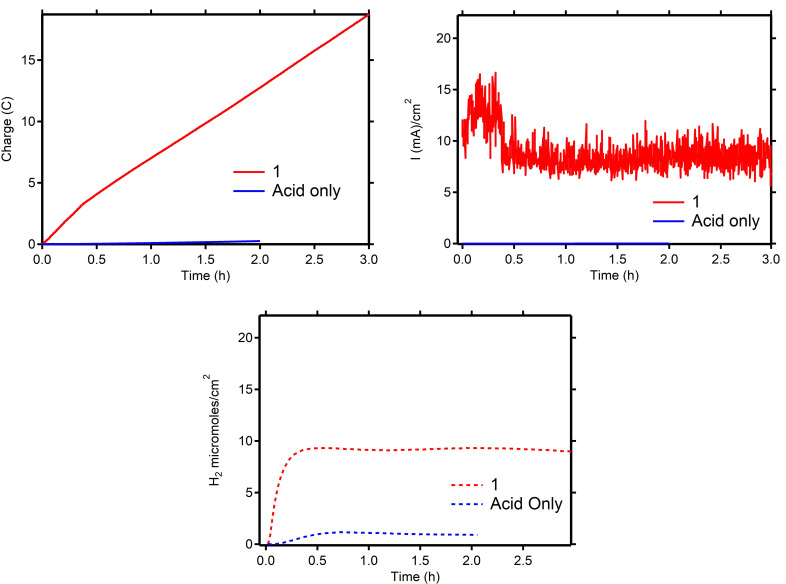
Top left: charge vs. time. Top right: current density vs. time. Bottom: hydrogen production vs. time. During CPE in DMF (0.1 m *n*Bu_4_NBF_4_ and 0.1 m Et_3_NHBF4) at −2.00 V versus Fc^+^/Fc at 0 rpm. Blue trace: control experiment in the absence of **1**. red trace: 1 mm **1**. Working electrode: glassy carbon RDE; counter‐electrode: Pt wire; reference electrode: Ag/Ag(NO_3_). H_2_ quantification was determined by in‐line GC measurements.

Based on our findings in the CV studies, controlled‐potential electrolysis (CPE) was performed at −2.00 V vs. Fc^+^/Fc. The reported thermodynamic reduction potential *E*°_HA_ of the Et_3_NHBF_4_ proton source in DMF is −1.31 V vs. Fc^+^/Fc, corresponding to an overpotential of 690 mV at the applied potential.^[17]^ During 3 h electrolysis of a 1.0 mm (bright pink) solution of **1** in DMF (10 mL) containing 0.1 m Et_3_NHBF_4_ and 0.1 m *n*Bu_4_NBF_4_ (supporting electrolyte) solution at 0 rpm, a relatively stable current with an average current density of 8.7 mA cm^−2^ (total charge of 18.7 C) and >99 % Faradaic efficiency (FE) was achieved (Table S1 and Figure 4). A control experiment in the absence of **1** delivered only 0.3 C after 2 h. These results demonstrate that **1** is catalytically activity for HER during CPE under these conditions with high selectivity.

The catalyst solution did not change color over the course of the electrolysis experiment, and ^1^H‐NMR analysis furthermore confirmed the presence of **1** as the major paramagnetic species in solution after electrolysis. This is in contrast with our previously reported bulk electrolysis experiments in MeCN using a mercury electrode, which showed that **1** is not stable for longer times (2 h) at an applied potential of −1.9 V vs. Fc^+^/Fc in MeCN.^[13]^ Next, our novel setup (with the glassy carbon cathode) prompted us to re‐evaluate the activity of **1** during CPE in MeCN solution with AcOH as proton source. The bulk solution did not show any optical changes over the course of the experiment, but interestingly also no catalytic activity was observed for **1** under these conditions (Figure S11). We therefore conclude that the stability and activity of **1** under CPE conditions is sensitive to both the electrode material and the solvent. Accordingly, we have continued our studies using DMF as the reaction solvent in combination with a glassy carbon electrode.

Our CV experiments indicated a diffusion‐controlled reductive process for **1** near the glassy carbon electrode surface, which encouraged us to enhance the substrate and catalyst delivery (mass transfer) towards the electrode by rotating the working electrode. In addition, the accumulating of H_2_ bubbles under the electrode surface was diminished upon rotation. CPE at 2000 rpm at −2.00 V vs. Fc^+^/Fc in DMF (1 mm **1**, 0.1 m Et_3_NHBF_4_ and 0.1 m *n*Bu_4_NBF_4_) showed a significant enhancement in catalytic performance (Table 1 and Figure 6, pink trace). During 3 h electrolysis, an average current density of 23.8 mA cm^−2^ (total charge 50.4 C), with an overall FE of >99 % was observed, which is a 2.5‐fold increase in activity without any loss of the quantitative FE. Control experiments in the absence of **1** did show significantly lower amounts of H_2_ being formed (Table 1 and Figure 6, black trace) and experiments in the absence of acid did not yield any hydrogen gas at all. These results denote the electrocatalytic HER rate of **1** can be increased upon better mass‐transfer.

**Table 1:** Bulk electrolysis experiments for H_2_ evolution using molecular Co^II^ complexes **1**–**4**.^[a]^

Catalyst (1 mm)	Charge [C]	Current density [mA cm^−2^]	FE [%]
–	3.8±0.5^[b]^	1.8	98^[b]^
1	50.4±0.7^[c]^	23.8	>99^[c]^
2	40.9±0.2^[c]^	19.3	95^[c]^
3	17.1	8.1	92
4	72.4±2.1^[c]^	34.2	95^[c]^

[a] Reaction conditions: 10 mL DMF, 0.1 m *n*Bu_4_NBF_4_, 0.1 m Et_3_NHBF_4_ at −2.00 V vs. Fc^+^/Fc, glassy carbon RDE, 2000 rpm, 3 h. H_2_ quantification by GC analysis. [b] Average of four measurements. [c] Average of two measurements.

**Figure 5:**
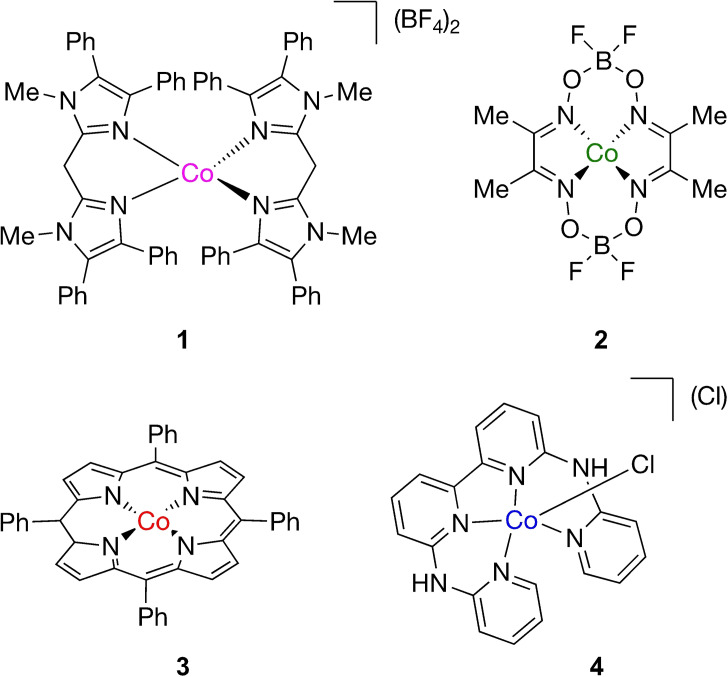
Molecular HER complexes **1**–**4**.

Next, the HER performance of **1** was benchmarked against three reported molecular cobalt(II) HER catalysts, including [Co(dmgBF_2_)_2_(solv)_2_] (**2**)^[21]^ (dmgBF_2_=difluoroboryldimethylglyoximato), [Co(TPP)] (**3**)^[22]^ (TPP=5,10,15,20‐tetraphenylporphyrinato), and [Co(bapbpy)Cl](Cl) (**4**)^[23]^ [bapbpy=6,6′‐bis‐(2‐aminopyridyl)‐2,2′‐bipyridine] (Figure 5). CVs of **2**–**4** were taken in DMF and matched those reported in the literature, showing catalytic activity in the presence of Et_3_NHBF_4_ (Figures S12–S14).

**Figure 6:**
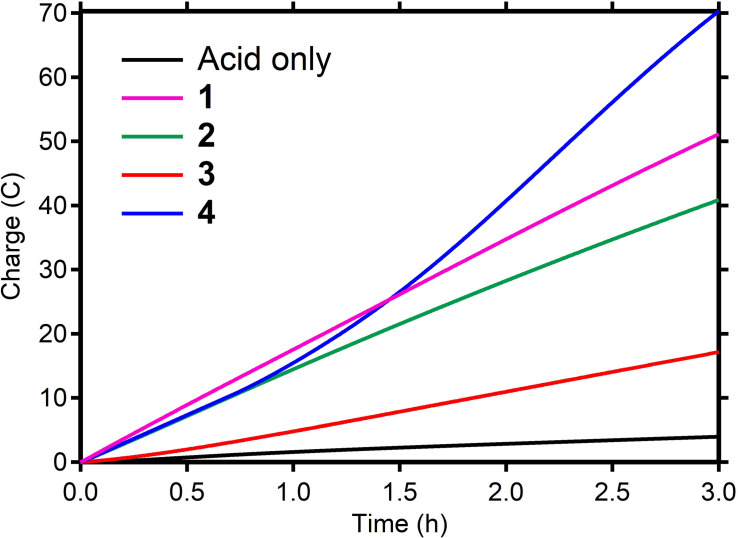
Rotating disk CPE: charge passed over the course of a bulk electrolysis experiment performed in the presence of 1 mm catalyst; pink: **1**; green: **2**; red: **3**; blue: **4**; black: no catalyst; 0.1 m Et_3_NHBF_4_ in DMF (containing 0.1 m *n*Bu_4_NBF_4_ as the supporting electrolyte) at −2.00 V vs. Fc^+^/Fc at 2000 rpm.

Complexes **2**–**4** were then tested under the same rotating disk CPE conditions as described for **1** (Table 1 and Figure 6), and all demonstrated catalytic proton reduction with average current densities (mA cm^−2^) increasing from 8.1 for **3**, 19.3 for **2**, and 34.2 for **4**. CPE performances were reproducible over several measurements. A closer look at the performances reveals that the selected conditions yield high FE (>92 %) for H_2_ for all complexes. In addition, a linear charge increase (equivalent to a stable current density) was observed for complexes **1**–**3**, which indicates a steady HER performance. Complex **4** is most active under these conditions but shows an increase in current density as well as hydrogen formation after 45 min. This observation is reproducible over two measurements and seems to indicate a structural change to **4** during catalysis that is not yet understood (Figure S15; see below). Complex concentrations near the electrode and diffusion coefficients are assumed to be similar between experiments, and therefore the current densities obtained for these 3 h experiments give an insight in the relative rate of the proton reduction for complexes **1**–**4** under the same conditions. The strong variations in performance demonstrate that the choice of molecular cobalt(II) complex (structure, overall charge, ligand) has a significant influence on the overall proton reduction performance under the applied conditions, with complex **1** showing a stable performance, which is second best in this series.

### Catalytic overpotential

**Figure 7:**
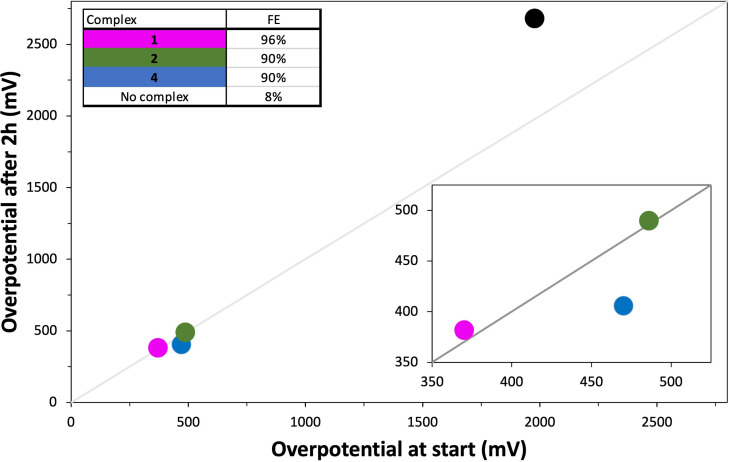
Plot of the overpotential requirement and stability for HER complexes **1** (pink), **2** (green), **4** (blue), and no complex (black) during controlled potential electrolysis in 10 mL DMF, 0.1 m *n*Bu_4_NBF_4_, 0.1 m Et_3_NHBF_4_ at −2.00 V vs. Fc^+^/Fc, glassy carbon RDE, 2000 rpm, 3 h. H_2_ quantification by GC analysis. The *x*‐axis represents the overpotential required to achieve 10 mA cm^−2^ per geometric area at time *t*=0. The *y*‐axis represents the overpotential required to achieve 10 mA cm^−2^ per geometric area at time *t*=2 h. The diagonal gray line is the expected response for a stable catalyst that does not change in activity during 2 h constant polarization. The region of interest for benchmarking is expanded in the inset plot.

In a next set of experiments, we investigated the overpotential requirement during a series of controlled current electrolysis (CCE) experiments for a current density of 10 mA cm^−2^, an amount relevant for the cathodic side of a solar water splitting device.^[5]^ The three most active complexes under above CPE conditions, **1**, **2**, and **4** were tested in a series of 2 h CCE experiments under the same conditions as the CPE experiments (1 mm **1**, **2**, or **4**; 0.1 m Et_3_NHBF_4_; 0.1 m *n*Bu_4_NBF_4_) and product formation was again measured by in‐line GC‐TCD analysis. To determine the overpotential, the dynamic voltages were recorded and subtracted from the reported thermodynamic reduction potential for Et_3_NHBF_4_ in DMF.^[17]^ The overpotential requirements at *t*=0 and *t*=2 h, shown in Figure 7, illustrate that the bare glassy carbon electrode (black) requires an overpotential around 2000 mV to reach the necessary current density with a poor FE of 8 %. In contrast, **1** (pink) has an overpotential of 370 mV at *t=*0 which slowly increases over time to 383 mV at *t=*2 h, with 96 % FE. **2** (green) requires a larger overpotential, starting at 486 mV and moving to 490 mV at *t=*2 h, with 90 % FE. Complex **4** (blue) shows an overpotential at *t=*0 of 470 mV, which surprisingly decreases overtime to 406 mV, meaning that less energy is required at *t=*2 h (FE=90 %). This decrease is in line with the rate increase observed during the CPE. Under the applied conditions, **1** performs best by requiring the lowest overpotential at *t=*0 as well as at *t=*2 h, combined with the highest FE.

### Post‐electrolysis analysis

Whereas no color changes were observed for the reaction solutions during CPE and CCE experiments, distinct optical changes to the glassy carbon working electrode surface were observed after electrocatalytic electrolysis experiments with all four complexes **1**‐**4**; that is, the electrode surface was covered with a thin layer of black deposit. To obtain insight in the formation of these deposits and their role in electrocatalysis, the deposits formed in the presence of molecular complexes **1** and **2** were further investigated.

**Figure 8:**
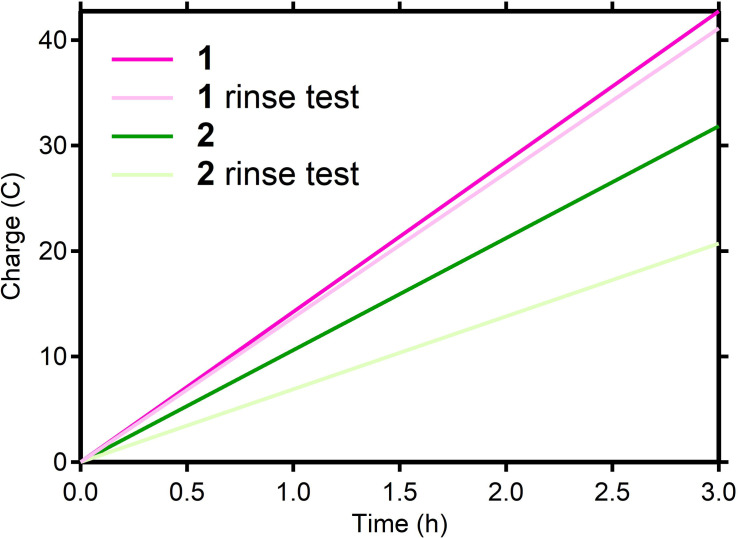
Charge passed over the course of 3 h controlled potential electrolysis performed in 0.1 m Et_3_NHBF_4_ in DMF (containing 0.1 m *n*Bu_4_NBF_4_ as the supporting electrolyte) at −2.00 V vs. Fc^+^/Fc at 2000 rpm. Ultra‐pink trace 0.5 mm **1** (pristine electrode), rose pink trace subsequent electrolysis with no homogeneous molecular complex **1** (modified electrode). Deep green trace 0.5 mm **2** (pristine electrode), bright‐green traces subsequent electrolysis with no homogeneous molecular catalyst **2** (modified electrode).

First, the catalytic HER activity of the deposit derived from **1** was investigated by a rinse test. A pristine glassy carbon RDE was put in a 0.5 mm solution of **1** and an CPE experiment was performed (Table 2, entry 1, Figure 8, pink trace). After 3 h, the pink reaction solution was removed from the electrocatalytic cell, while the electrode was kept under inert conditions. Next, the working electrode compartment was washed with 10 mL dry DMF, followed by the addition of a fresh reaction solution without **1**. Visual inspection did not indicate any physical changes to the working electrode, including its surface modifications, during the washing and replacement of the reaction medium, which suggests that the deposition is not merely an electrostatic interaction of the dicationic cobalt complex with the cathode when a cathodic potential is applied. Finally, another CPE experiment was carried out (Figure 8, light pink trace and Table 2, entry 2), showing that the deposition on the electrode surface is an active catalytic material for proton reduction. Monitoring the activity of the deposit overtime shows that the current density decreases over time (Figure S16). Over 3 h, a current density average of 19.4 mA cm^−2^ with a quantitative FE was observed, which is a slight decrease in performance compared to the initial CPE in the presence of **1**. The same rinse test experiment was performed for **2** (Figure 8, green traces; Table 2, entries 3 and 4). The rinse test for **2** also demonstrated catalytic activity for the deposit, albeit with a significantly decreased current density (initial: 15.0 mA cm^−2^, rinse test: 9.8 mA cm^−2^).

**Table 2:** Rinse test HER electrolysis.^[a]^

Entry	Catalyst (0.5 mm)	Charge [C]	Current density [mA cm^−2^]	FE [%]
1	1	42.7	20.2	98
2	rinse test	41.1	19.4	>99
3	2	31.8	15.0	97
4	rinse test	20.7	9.8	99

[a] Reaction conditions: 10 mL DMF, 0.1 m *n*Bu_4_NBF_4_, 0.1 mm Et_3_NHBF_4_ at −2.00 V vs. Fc^+^/Fc, glassy carbon RDE, 2000 rpm, 3 h. H_2_ quantification by GC analysis.

Whereas the rinse tests clearly show electrocatalytic HER activity for the deposits formed from **1** and **2**, differences in the overall activity of the deposits were observed. This observation suggests that putative decomposition of the molecular complexes does not lead to the same deposit, that is, the molecular complex governs the activity of the deposit, either by leading to a different composition or a different structure of the deposit. More importantly, these results indicate that electrocatalytic HER activity during CPE by the complexes used in our investigations is not merely a feature of the molecular complex in solution but is to a large extent determined by a deposit formed during the CPE experiment.

### Analysis of the deposit

To gain information on the structure and composition of the electrode deposits formed during CPE, post‐electrolysis X‐ray photoelectron spectroscopy (XPS) analysis of the deposit formed from **1** was performed. The elements Co, B, F, N, O, and C were found to be present in the deposit, in relative atomic amounts of 0.23, 5.0, 15.7, 4.04, 4.29, and 68.4 %, respectively (Figure S17). Firstly, these atomic percentages indicate that **1** (Co 4.98 %, B 1.81 %, F 12.73 %, N 9.39 %, C 66.41 %) does not simply absorb on the electrode surface, nor that **1** is completely reduced to metallic cobalt. Instead, the surface is covered with a combination of atoms originating from **1**, the DMF solvent, the electrolyte salt, and/or the sacrificial proton source. Important to note is that the high contribution of carbon is caused by the usage of carbon tape to support the sample. The 4.29 % oxygen most likely originates from the DMF solvent and air exposure before XPS analysis as no other oxygen source was added during electrolysis. Nitrogen could originate from all components in the reaction medium. Fluor and boron signals can originate from both the electrolyte salt and the counterions of **1**, although in both cases a relative ratio closer to 4 : 1 would have been expected. Cobalt is with 0.23 % only a minor contributor to the deposition.

A closer look at the cobalt signal (Figure S18) revealed a broad signal for the 2p_3/2_ core level of Co 2p with several contributions (i. e., at 781.2 and 786.0 eV, and a minor contribution at 777.4 eV). The former values can be assigned to Co^II^ species, while the latter corresponds to metallic cobalt (see the work of Biesinger and co‐workers^[24c]^ for the deconvolution of the Co spectrum).[^11b^, ^24^] Hence it became clear that most of the absorbed cobalt remains in the 2^+^ oxidation state, although some reduced cobalt is present. Due to the low amount of Co in the deposit, the amount of metallic Co could not be resolved reliably. Further studies will have to provide information on whether **1** maintains its structural integrity and is somehow incorporated in the deposit, or that it decomposes.

Literature analysis shows some precedence for the formation of deposits during non‐aqueous HER experiments using molecular cobalt complexes.[^12c^, ^12e^, ^12g^, ^24a^, ^25^] A dicationic tris(glyoximato) cobalt clathrochelate was reported to form a deposit during HER electrolysis in MeCN with NaClO_4_ as supporting electrolyte salt and HClO_4_ as sacrificial proton donor. In this case, energy‐dispersive X‐ray spectroscopy (EDX) analysis showed a relative ratio of atomic amounts adsorbed to the electrode of 63±7 % : 32.5±5 % cobalt/heteroatoms (F, O, N, B), which significantly deviates from our observations, particularly in terms of the amount of cobalt.^[12e]^

## Conclusion

Voltammetry investigations have shown that cobalt complex **1** has a more reversible Co^II^/Co^I^ couple in DMF, as compared to the irreversible behavior in MeCN observed in our previous studies. Accordingly, its electrochemical solution‐state behavior and electrocatalytic performance could be analyzed in more detail in DMF during voltammetry. **1** was found to be an active hydrogen evolution reaction (HER) electrocatalyst in the presence of a proton donor. In bulk electrolysis experiments in the presence of triethylammonium, **1** is a competitive HER catalyst amongst a benchmarking pool of four molecular cobalt HER catalysts, including the widely studied cobalt dimethylglyoxime complex. Bulk electrolysis experiments (controlled potential and controlled current electrolysis) also showed the formation of a deposit on the carbon electrode during electrolysis for all complexes tested, including **1**. Rinse experiments showed that deposits formed from **1** and **2** have only a somewhat decreased HER activity in a next electrolysis experiment. These observations indicate that, under the current circumstances, all molecular complexes tested in this study (including **1**) seem to act as pre‐catalysts that form a heterogenous material (electrode deposit) responsible for HER activity. Importantly, the deposit formed from **1** has a significantly different chemical composition than **1**, based on X‐ray photoelectron spectroscopy analysis, with a small but significant percentage of Co (0.23 %) composed mostly of Co^II^ and with a minor contribution of Co^0^. This is in contrast with several earlier studies that observed mainly metallic cobalt in their deposits.

With this study, we have shown that the observed catalytic performance for **1** during electrocatalytic voltammetry is distinct from its bulk electrolysis performance due to physical changes at the electrode surface by means of a deposit formation during bulk electrolysis. Similar observations were also made for a series of other molecular, Co‐based HER electrocatalysts. Interestingly, our studies showed that the molecular design of the catalysts influence the composition of the deposit and therewith the observed activity in our model electrolysis cell. These findings are of interest for the development of molecular HER catalysts and of immobilized electrocatalytic materials.

## Experimental Section

Typical electrocatalytic controlled potential and current experiments were carried out in a two‐compartment three‐electrode electrochemical cell (Figure S10). First, the electrochemical cell was put in a glovebox, where the counter electrode (CE) compartment was filled with 10 mL electrolyte solution, the double junction reference electrode (RE) was filled with a 0.01 m solution of Ag/Ag(NO_3_) and the working electrode (WE) compartment was filled with a pre‐mixed solution of electrolyte, sacrificial proton donor, and molecular complex. Then, the gas inlet and outlet were closed with rubber stoppers, the CE and RE were positioned on the outer shafts, and a glass stopper was put on the WE shaft. Then, the electrochemical cell was transferred out of the glovebox and put under a flow of N_2_ (5 mL min^−1^), which was in‐line with the GC apparatus. Subsequently, the RDE was replaced for a glass stopper and the cell was purged until the residual oxygen signal (originating from the assembly of the cell outside of the glovebox) completely disappeared on the GC chromatograms (Figure S19), which usually took 5–10 min. Finally, electrolysis was performed as described.

## Author Contributions

Synthesis, characterization and electrochemical measurements were done by S. D. de Vos and M. Otten. XPS analysis was done by T. Wissink and E. J. M. Hensen. Project design was done by S. D. de Vos and R. J. M. Klein Gebbink. Funding acquisition, administration and oversight were done by R. J. M. Klein Gebbink. The original draft was written by S. D. de Vos and reviewing and editing was done by D. L. J. Broere, R. J. M. Klein Gebbink with contributions by all authors.

## Conflict of interest

The authors declare no conflict of interest.

1

## Supporting information

As a service to our authors and readers, this journal provides supporting information supplied by the authors. Such materials are peer reviewed and may be re‐organized for online delivery, but are not copy‐edited or typeset. Technical support issues arising from supporting information (other than missing files) should be addressed to the authors.

Supporting Information

## Data Availability

The data that support the findings of this study are available in the supplementary material of this article.

## References

[1] N. S. Lewis , D. G. Nocera , Proc. Natl. Academy Sci. USA 2006, 103, 15729–15735. 10.1073/pnas.0603395103 PMC1635072 17043226

[2] I. Roger , M. A. Shipman , M. D. Symes , Nat. Rev. Chem. 2017, 1, 0003.

[3] S. Trasatti , J. Electroan. Chem. Interfacial Electrochem. 1972, 39, 163–184.

[4] R. M. Bullock , J. G. Chen , L. Gagliardi , P. J. Chirik , O. K. Farha , C. H. Hendon , C. W. Jones , J. A. Keith , J. Klosin , S. D. Minteer , R. H. Morris , A. T. Radosevich , T. B. Rauchfuss , N. A. Strotman , A. Vojvodic , T. R. Ward , J. Y. Yang , Y. Surendranath , Science 2020, 369, eabc3183. 32792370 10.1126/science.abc3183 PMC7875315

[5] C. C. L. McCrory , S. Jung , I. M. Ferrer , S. M. Chatman , J. C. Peters , T. F. Jaramillo , J. Am. Chem. Soc. 2015, 137, 4347–4357. 25668483 10.1021/ja510442p

[6a] L. Chen , M. Wang , K. Han , P. Zhang , F. Gloaguen , L. Sun , Energy Environ. Sci. 2014, 7, 329–334;

[6b] M. L. Helm , M. P. Stewart , R. M. Bullock , M. R. DuBois , D. L. DuBois , Science 2011, 333, 863–866; 21836012 10.1126/science.1205864

[6c] K. Koshiba , K. Yamauchi , K. Sakai , Angew. Chem. Int. Ed. 2017, 56, 4247–4251. 10.1002/anie.201700927 28276659

[7] J. R. McKone , S. C. Marinescu , B. S. Brunschwig , J. R. Winkler , H. B. Gray , Chem. Sci. 2014, 5, 865–878.

[8a] V. Artero , M. Chavarot-Kerlidou , M. Fontecave , Angew. Chem. Int. Ed. 2011, 50, 7238–7266; 10.1002/anie.201007987 21748828

[8b] N. Queyriaux , R. T. Jane , J. Massin , V. Artero , M. Chavarot-Kerlidou , Coord. Chem. Rev. 2015, 304–305, 3–19; 10.1016/j.ccr.2015.03.014 PMC4681115 26688590

[8c] J. L. Dempsey , B. S. Brunschwig , J. R. Winkler , H. B. Gray , Acc. Chem. Res. 2009, 42, 1995–2004; 19928840 10.1021/ar900253e

[8d] D. Dolui , S. Ghorai , A. Dutta , Coord. Chem. Rev. 2020, 416, 213335;

[8e] G. C. Tok , A. T. S. Freiberg , H. A. Gasteiger , C. R. Hess , ChemCatChem 2019, 11, 3973–3981;

[8f] M. M. Roubelakis , D. K. Bediako , D. K. Dogutan , D. G. Nocera , Energy Environ. Sci. 2012, 5, 7737–7740.

[9a] K. J. Lee , B. D. McCarthy , J. L. Dempsey , Chem. Soc. Rev. 2019, 48, 2927–2945; 31089606 10.1039/c8cs00851e

[9b] V. Artero , M. Fontecave , Chem. Soc. Rev. 2013, 42, 2338–2356. 23165230 10.1039/c2cs35334b

[10] J.-M. Savéant , Elements of molecular and biomolecular electrochemistry: an electrochemical approach to electron transfer chemistry, John Wiley & Sons, 2006.

[11a] J. A. Widegren , R. G. Finke , J. Mol. Catal. A 2003, 198, 317–341;

[11b] N. Kaeffer , A. Morozan , J. Fize , E. Martinez , L. Guetaz , V. Artero , ACS Catal. 2016, 6, 3727–3737.

[12a] E. Anxolabéhère-Mallart , C. Costentin , M. Fournier , M. Robert , J. Phys. Chem. C 2014, 118, 13377–13381;

[12b] D. J. Martin , B. D. McCarthy , C. L. Donley , J. L. Dempsey , Chem. Commun. 2015, 51, 5290–5293; 10.1039/c4cc08662g 25470993

[12c] D. J. Sconyers , J. D. Blakemore , Chem. Commun. 2017, 53, 7286–7289; 10.1039/c7cc02188g 28426091

[12d] B. D. McCarthy , C. L. Donley , J. L. Dempsey , Chem. Sci. 2015, 6, 2827–2834; 29403633 10.1039/c5sc00476d PMC5761499

[12e] E. Anxolabéhère-Mallart , C. Costentin , M. Fournier , S. Nowak , M. Robert , J.-M. Savéant , J. Am. Chem. Soc. 2012, 134, 6104–6107; 22458714 10.1021/ja301134e

[12f] S. El Ghachtouli , M. Fournier , S. Cherdo , R. Guillot , M.-F. Charlot , E. Anxolabéhère-Mallart , M. Robert , A. Aukauloo , J. Phys. Chem. C 2013, 117, 17073–17077;

[12g] S. E. Ghachtouli , R. Guillot , F. Brisset , A. Aukauloo , ChemSusChem 2013, 6, 2226–2230. 24155088 10.1002/cssc.201300564

[13] P. Ghosh , S. de Vos , M. Lutz , F. Gloaguen , P. Schollhammer , M.-E. Moret , R. J. M. Klein Gebbink , Chem. Eur. J. 2020, 26, 12560–12569. 32350932 10.1002/chem.201905746 PMC7589288

[14] K. J. Lee , N. Elgrishi , B. Kandemir , J. L. Dempsey , Nat. Rev. Chem. 2017, 1, 0039.

[15a] N. Elgrishi , D. A. Kurtz , J. L. Dempsey , J. Am. Chem. Soc. 2017, 139, 239–244; 27997157 10.1021/jacs.6b10148

[15b] E. S. Wiedner , R. M. Bullock , J. Am. Chem. Soc. 2016, 138, 8309–8318; 27300721 10.1021/jacs.6b04779

[15c] N. Elgrishi , M. B. Chambers , V. Artero , M. Fontecave , Phys. Chem. Chem. Phys. 2014, 16, 13635–13644. 24651983 10.1039/c4cp00451e

[16] D. J. Graham , Standard Operating Procedures for Cyclic Voltammetry, Lulu Press, Incorporated, 2017.

[17] G. A. N. Felton , R. S. Glass , D. L. Lichtenberger , D. H. Evans , Inorg. Chem. 2006, 45, 9181–9184. 17083215 10.1021/ic060984e

[18] V. Fourmond , P.-A. Jacques , M. Fontecave , V. Artero , Inorg. Chem. 2010, 49, 10338–10347. 20964310 10.1021/ic101187v

[19] R. M. Bullock , A. M. Appel , M. L. Helm , Chem. Commun. 2014, 50, 3125–3143. 10.1039/c3cc46135a 24448464

[20a] J. Tafel , Z. Phys. Chem. 1905, 50 U, 641–712;

[20b] J.-M. Savéant , Chem. Rev. 2008, 108, 2348–2378. 18620367 10.1021/cr068079z

[21] M. Razavet , V. Artero , M. Fontecave , Inorg. Chem. 2005, 44, 4786–4795. 15962987 10.1021/ic050167z

[22] B. B. Beyene , S. B. Mane , C.-H. Hung , J. Electrochem. Soc. 2018, 165, H481-H487.

[23] N. Queyriaux , D. Sun , J. Fize , J. Pécaut , M. J. Field , M. Chavarot-Kerlidou , V. Artero , J. Am. Chem. Soc. 2020, 142, 274–282. 31760743 10.1021/jacs.9b10407

[24a] S. Cobo , J. Heidkamp , P.-A. Jacques , J. Fize , V. Fourmond , L. Guetaz , B. Jousselme , V. Ivanova , H. Dau , S. Palacin , M. Fontecave , V. Artero , Nat. Mater. 2012, 11, 802–807; 22863815 10.1038/nmat3385

[24b] S. Hüfner , Photoelectron spectroscopy: principles and applications, Springer Science & Business Media, 2013;

[24c] M. C. Biesinger , B. P. Payne , A. P. Grosvenor , L. W. M. Lau , A. R. Gerson , R. S. C. Smart , Appl. Surface Sci. 2011, 257, 2717–2730.

[25] X. L. Ho , S. P. Das , L. K.-S. Ng , A. Y. R. Ng , R. Ganguly , H. S. Soo , Organometallics 2019, 38, 1397–1406.

