# Supplementary material for: Hydrogen Evolution Electrocatalysis with a Molecular Cobalt Bis(alkylimidazole)methane Complex in DMF: a Critical Activity Analysis
Source: ChemSusChem. 2022 Oct 26;15(22):e202201308. doi: 10.1002/cssc.202201308 (PMC9828534; doi:10.1002/cssc.202201308)
Supplement: Supplementary file 1 — Supporting Information [file CSSC-15-0-s001.pdf]

ChemSusChem

Supporting Information

Hydrogen Evolution Electrocatalysis with a Molecular Cobalt Bis(alkylimidazole)methane Complex in DMF: a Critical Activity Analysis

Sander D. de Vos, Maartje Otten, Tim Wissink, Daniël L. J. Broere, Emiel J. M. Hensen, and Robertus J. M. Klein Gebbink*© 2022 The Authors. ChemSusChem published by Wiley-VCH GmbH. This is an open access article under the terms of the Creative Commons Attribution License, which permits use, distribution and reproduction in any medium, provided the original work is properly cited.

Table of contents

General Information and Experimental Procedures S2-S3

List of Figures

Figure S1	CV of **1** at different scan rates in MeCN	S4
Figure S2	CV of **1** at different scan rates in DMF	S4
Figure S3	Peak current analysis of **1**	S5
Figure S4	^{1}H NMR of **1** in DMF-d_7 and MeCN-d_3	S6
Figure S5	^{1}H-NMR of 1 in DMF-d_7 + 0, 10 and 100 equiv. Et_3NHBF_4	S6
Figure S6	Proportional catalytic peak currents as a function of the acid concentration	S7
Figure S7	Proportional catalytic peak currents as a function of the square root of the scan rate	S7
Figure S8	CV of **1** in DMF + 5 equiv. Phenol	S8
Figure S9	CV of Et_3NHBF_4 only in DMF	S8
Figure S10	Electrolysis HER Model-Cell Design	S9
Figure S11	Charge consumed during CPE in the presence/absence of **1** in MeCN + AcOH	S10
Figure S12	CVs of [Co(dmgBF_2)$_2$(solv)$_2$] (**2**) (+ 1 equiv. Et_3NHBF_4) recorded in DMF	S11
Figure S13	CVs of [Co(TPP)] (**3**) (+ 1 equiv. Et_3NHBF_4) recorded in DMF	S11
Figure S14	CVs of [Co(bapbpy)Cl](Cl) (**4**) (+ 1 equiv. Et_3NHBF_4) recorded in DMF	S11
Figure S15	Hydrogen production of **4** during CPE	S12
Figure S16	Raw data of rinse test experiments with **1** in DMF during CPE	S13
Figure S17	XPS spectrum of the electrode deposit of **1**	S13
Figure S18	XPS spectrum of the electrode deposit of **1** Co region	S14
Figure S19	GC-TCD chromatograms	S14
Figure S20	GC-TCD calibration with H_2/N_2 mixtures	S15

List of Tables

Table S1	Stationary bulk electrolysis with **1** in DMF	S10

General information and experimental procedures

General procedures

All air-sensitive reactions were carried out under an inert atmosphere of water- and oxygen-free N_2 gas using standard Schlenk techniques or were performed in an MBraun labmaster dp glovebox workstation. Dry acetonitrile (MeCN), dichloromethane (CH_2Cl_2) and methanol (MeOH) were used from an MBraun SPS-800 solvent purification system, dried over 3 or 4 Å molecular sieves and degassed by bubbling N_2 for at least 30 min. Tetrahydrofuran (THF) was dried over sodium benzophenone after taking it from an MBraun SPS-800 solvent purification system, subsequently distilled and degassed by bubbling N_2 for at least 30 min. Anhydrous *N,N*-Dimethylformamide (DMF) was purchased from Sigma-Aldrich and subsequently dried over 4 Å molecular sieves and degassed by bubbling N_2 for at least 30 min. All other commercially obtained chemicals were used without further purification, unless stated otherwise. ^{1}H-NMR and ^{13}C-NMR spectra were recorded at 400 MHz, 101 MHz and 376 MHz respectively, at 298 K, on a Varian VNMRS400 or an Oxford NMR AS400 spectrometer. Chemical shifts (δ) are reported in ppm and referenced against the residual solvent signal. XPS was performed with a K-Alpha XPS apparatus (Thermo Scientific). An aluminum anode was used as the X-Ray source (Kα monochromatic irradiation, 1486.6 eV) operating at 72 W and a spot size of 400 µm. High-resolution regional spectra were measured with a pass energy of 50 eV and wide-range survey spectra were recorded at 200 eV pass energy. The pressure inside the analysis chamber was kept below a maximum of $3\cdot10^{-7}$ mbar during the measurements. Charge neutralization was applied by a low-energy Ar+ ion beam. The spectra were analyzed using the CasaXPS software.

Electrochemical sweeping experiments were performed in a nitrogen-filled MBraun labmaster dp glovebox, on an IVIUM potentiostat/galvanostat using a 3-electrode setup including a Pt-wire counter electrode, a glassy carbon-working electrode (3 mm Ø) and an Ag/Ag(NO_3) reference electrode used directly in solution. The potentiostat was kept external to the glovebox, and the electrode leads were connected with a custom shielded electrode cable feedthrough. All scans were absolutely referenced to the ferrocenium/ferrocene redox couple and taken at a scan rate of 100 mV.s^{-1}, unless reported differently. The working electrodes were polished with 0.3 µm aluminum oxide powder deionized water slurries and rinsed with water for 30 seconds to remove residual polishing powder. All experiments were performed on clear and non-turbid solitons. Before all experiments, background voltammograms were recorded at a scan rate of 100 mV.s^{-1}. Solutions were mixed by shaking before each measurement and the working electrode surface was cleaned with a tissue after every scan. All measurements were recorded in a 0.1 M tetrabutylammonium tetrafluoroborate (*n*Bu_4NBF_4) solution in dry, degassed DMF or MeCN. Electrolysis solutions were prepared in the glovebox and electrolysis experiments were performed outside the glovebox using a Autolab PGSTAT204 potentiostat/galvanostat, using a 3-electrode setup including a Pt-plate counter electrode, a RDE glassy carbon-working electrode (5 mm Ø) and a double junction Ag/Ag(NO_3) reference electrode used directly in solution. All electrodes for electrolysis experiments were purchased from Methrom. For details on the electrochemical cell for electrolysis experiments, see below.

Synthesis

[Co(HBMIM^{Ph2})$_2$](BF_4)$_2$ (**1**), (HBMIMPh2 = bis(1-methyl-4,5-diphenyl-1H-imidazol-2-yl)methane) was synthesized according to our previously reported methods.[1] Dissolving **1** in MeCN or DMF (up to 10 mM) leads to clear and non-turbid pink solutions. All electrochemical measurements were performed at lower concentrations (0.5 – 2 mM)

Additional characterization in MeCN and DMF:
UV-Vis: d-d transitions: (MeCN ε [L mol^{-1} cm^{-1}]): λ_{max} 555 (440), 510 (410) nm. UV-Vis (DMF ε [L mol^{-1} cm^{-1}]): λ_{max} 555 (430), 505 (400) nm

^{1}H NMR (400 MHz, MeCN-d$_3$, 25 °C) (Figure S4 bottom): *δ* (ppm) = 182.82 (1H, CH_2), 28.26 (3H, CH_3), 11.69 (1H, Ph *p*-CH), 7.04 (2H, Ph *o*-CH), 5.03 (1H, Ph *p*-CH), 3.64 (1H, Ph *m*-CH), 2.85 (1H, Ph *m*-CH), 2.27 (2H, Ph *m*-CH), –13.74 (2H, Ph *o*-CH). Tentative peak assignments are based on relative integration, and broadness of the resonance (methylene and some *o*-Ph CH protons seem to be in close proximity to the metal center, as observed in a solid-state single crystal X-ray structure, and therefore broaden), chemical shift, and similarity to structural analogs. Some integrals deviate from the expected value, presumably due to the broadness of the resonance.

^{1}H NMR (400 MHz, (DMF-d$_7$), 25 °C) (Figure S4 top): *δ* (ppm) = 185.90 (1H, CH_2), 28.13 (3H, CH_3), 12.07 (1H, Ph *p*-CH), 7.44 (1H, Ph *m*-CH), 7.14 (2H, Ph *o*-CH), 5.05 (1H, Ph *p*-CH), 3.92 (1H, Ph *m*-CH), 3.65 (1H, Ph *m*-CH), 2.96 (1H, Ph *m*-CH), –12.34 (2H, Ph *o*-CH). Tentative peak assignments are based on relative integration, and broadness of the resonance (methylene and some *o*-Ph CH protons seem to be in close proximity to the metal center, as observed in a solid-state single crystal X-ray structure, and therefore broaden), chemical shift, and similarity to structural analogs. Some integrals deviate from the expected value, presumably due to the broadness of the resonance.

^{1}H NMR spectra in MeCN-d$_3$ and DMF-d$_7$ are depicted in Figure S4. All observed resonances were attributed to **1**. The observed resonances for **1** in these solvents closely match in terms of chemical shifts, broadness of the signals, and integration. Therefore, we propose that **1** in MeCN or DMF solution is one and the same species. This observation also excludes the coordination of MeCN or DMF.

[Co(dmgBF$_2$)$_2$(solv)]$_2$] (**2**), (dmgBF$_2$ = difluoroboryldimethylglyoximato), was synthesized according to a literature procedure.[2] Cyclic voltammograms in presence and absence of 1 equiv. Et_3NHBF_4 in DMF (0.1M *n*Bu_4NBF_4) are reported in Figure S12.

[Co(TPP)] (**3**), (TPP = 5,10,15,20-tetraphenylporphyrinato) was purchased from Sigma Aldrich. Cyclic voltammograms in presence and absence of 1 equiv. Et_3NHBF_4 in DMF (0.1M $n$$Bu_4NBF_4$) are reported in Figure S13.

[Co(bapbpy)Cl](Cl) (**4**), (bapbpy = 6,6'-bis-(2-aminopyridyl)-2,2'-bipyridine) was synthesized according to a literature procedure.[3] Cyclic voltammograms in presence and absence of 1 equiv. Et_3NHBF_4 in DMF (0.1M $n$$Bu_4NBF_4$) are reported in Figure S14.

Acid−base properties and stability factors

The stability of **1** in the presence of increasing amounts of a sacrificial proton source was determined with ^{1}H-NMR. We monitored **1** in the presence of increasing amounts of Et_3NHBF_4in DMF (pK_a = 9.2).[4] Upon the addition of 10 and 100 equiv. Et_3NHBF_4, as a solid acid, the solution containing the complex remained pink and no significant changes in paramagnetic chemical shifts and peak intensities relative to the residual solvent signal were observed (Figures S5 & S6). These observations show that **1** does not degrade nor gets protonated in the presence of substantial amounts of the acid used, which adds to our observations on its stability in the presence of the weaker acid AcOH in MeCN solution.[1]

Electrochemical measurements coupled to gas chromatography for H_2-evolution quantification

Controlled potential and current measurements were carried out in a two-compartment three-electrode electrochemical cell (Figure S10). Prior to use, the catalytic cell was placed in a 1 M HCl solution for 12 h, followed by extensive washing with demineralized water, then it was put in an oven at 70 °C for 2 h after which it was directly put under vacuum and entered in a O_2/H_2O-free glovebox. The rotating disk working electrode and reference electrodes were placed in the same compartment filled with electrolyte solution, sacrificial proton donor and a molecular cobalt complex. The counter electrode was placed in the second compartment of the cell filled with only electrolyte solution. Then the cell and electrodes are placed in-line with the GC apparatus. Subsequently, the system was flushed with N_2 for 5-10 min to removed residual oxygen, the rotator was put on 0 or 2000 rpm and electrolysis was started.

H_2 quantification was accomplished via an in-line gas chromatography (GC) configuration. The cell compartment of the working electrode was continuously flushed by N_2 carrier gas, whose rate was fixed through a Bronkhorst EL FLOW prestige mass flowmeter/controller at 5 mL·min^{-1}. The output gas was analyzed with a InterScience CompactGC$^{4.0}$ gas chromatograph equipped with a Rt-QBond 3m * 0.32mm capillary column and Molsieve 5 A 7 m *0.32 mm capillary column thermostated at 65 °C and a TCD detector thermostated at 110 °C. The GC was mounted in the so-called continuous flow mode in which the carrier gas stream fills an injection loop of 50 µL in the GC. The content of the injection loop was fed every 76 sec into the GC setup, where gases (H_2 and O_2) are separated and the area under the peaks computed. Calibration of the GC-TCD was done by flushing mixed gases of N_2 and H_2 with different concentration through the electrocatalytic cell (Figure S20). Under the applied flow (5 mL/min), no air-leakages were determined, as confirmed by the absence of an oxygen signal in the GC (Figure S19). The experimentally obtained values from the chromatogram were then used to determine the total production of H_2 in the electrochemical cell (η_{obs}).
The theoretically production rate for H_2 derived from the current i passed in the cell, assuming a unitary faradaic yield, was calculated following the formula: $\eta_{curr} = i/(2 \times F)$ where F is the Faraday constant (F = 96485 C·mol^{-1}). The η_{obs}/η_{curr} ratio then gives the instantaneous faradaic yield of the electrode for H_2-evolution.

Calculation of diffusion constants from cyclic voltammograms of **1**

The peak current i_p is described by the Randles-Sevcik equation 1.

$$i_p = 0.4463(F/RT)^{1/2} n_p^{3/2} FAD^{1/2}[Co]v^{1/2} \quad \text{(eq.1)}$$

In equation 1, i_p is peak current, F is Faraday's constant (F = 96500 C mol^{-1}), R is the universal gas constant (R = 8.31 J K^{-1} mol^{-1}), T is temperature (T = 298 K) n_p is the number of electrons transferred (n_p = 1 for Co(II)/Co(I)), A is the active surface area of the electrode (A = 0.07 cm^2), D is the diffusion coefficient for the complex, [Co] is the concentration of the catalyst, and v is the scan rate. The diffusion coefficient (D) was calculated from the i_{pc} of the reversible scans between 4000–20000 mV/s giving a value of 3.5 × 10^{-6} cm^2 s^{-1} (± 0.3 × 10^{-6}).

Calculation of Turn Over Frequency (TOF) from cyclic voltammograms

The TOF (K_{obs}) was determined using eq. 2, in which i_p is the peak current of the Co$^{II/I}$ couple in the absence of acid, i_{cat} the catalytic current in the presence of acid, n is the electron stoichiometry, R is the gas constant, T is the temperature in Kelvin, F is the Faraday constant in C/mol, v is the scan rate in V/s and k_{obs} is the observed rate constant.[5]

$$\frac{i_{cat}}{i_p} = \frac{n}{0.4463}\sqrt{\frac{RTk_{obs}}{Fv}} \quad \text{(eq.2)}$$

For a two-electron catalytic process, n = 2, at 25 °C, *Equation 2* can be simplified to:

$$k_{obs}(TOF) = 1.94 \cdot v \cdot \left(\frac{i_{cat}}{i_p}\right)^2 \quad \text{(eq.3)}$$

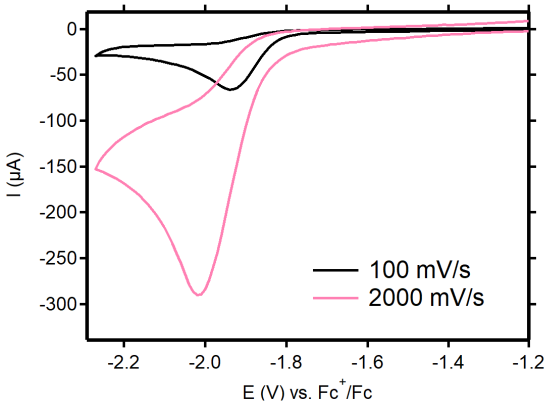

Figure S1. CV of **1** (2 mM) in MeCN (containing 0.1 M *n*Bu_4NBF_4 as supporting electrolyte) at different scan rates; 100 and 2000 mV.s^{-1}. Potentials in V vs Fc^+/Fc. Working electrode: Glassy carbon. Counter-electrode: Pt wire. Reference electrode: Ag/Ag(NO_3).

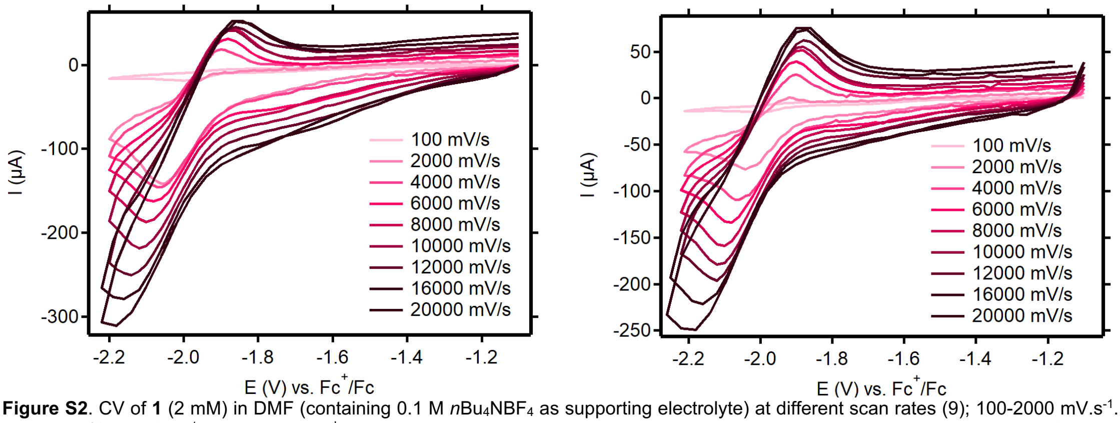

Figure S2. CV of **1** (2 mM) in DMF (containing 0.1 M *n*Bu_4NBF_4 as supporting electrolyte) at different scan rates (9); 100-2000 mV.s^{-1}. Potentials in V vs Fc^+/Fc. Left: 1st cycle, Right: 2nd consecutive cycle. Working electrode: Glassy carbon. Counter-electrode: Pt wire. Reference electrode: Ag/Ag(NO_3).

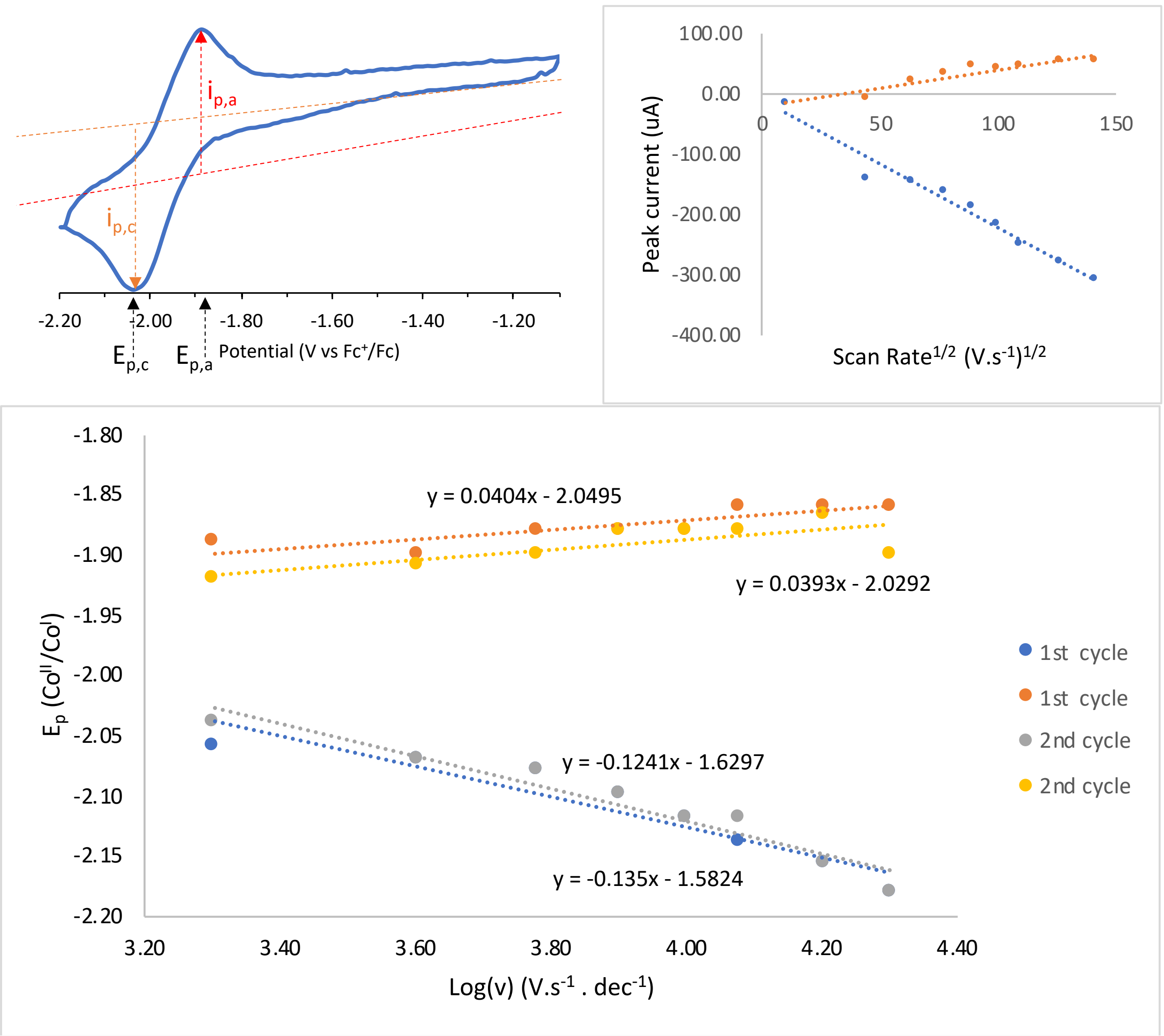

Figure S3. Top Left: Analysis of $i_{p,a}$, $i_{p,c}$, $E_{p,a}$, $E_{p,c}$ of **1** at 4000 mV/s. Top right: Plot of the anodic ($i_{p,a}$) and cathodic ($i_{p,c}$) peak currents for the Co(II)/Co(I) process of **1** versus the square-root of scan rate. Measurements performed in DMF (containing 0.1 M *n*Bu_4NBF_4 as supporting electrolyte). Bottom: Plot of the anodic ($E_{p,a}$) and cathodic ($E_{p,c}$) peak potentials for the Co(II)/Co(I) processes of **1** *versus* the square-root of scan rate. Measurements performed in DMF (containing 0.1 M *n*Bu_4NBF_4 as supporting electrolyte). Scan rate: 2000-20000 mV.s^{-1}. Potentials in V vs Fc^+/Fc. Working electrode: glassy carbon. Counter-electrode: Pt wire. Reference electrode: Ag/Ag(NO_3).

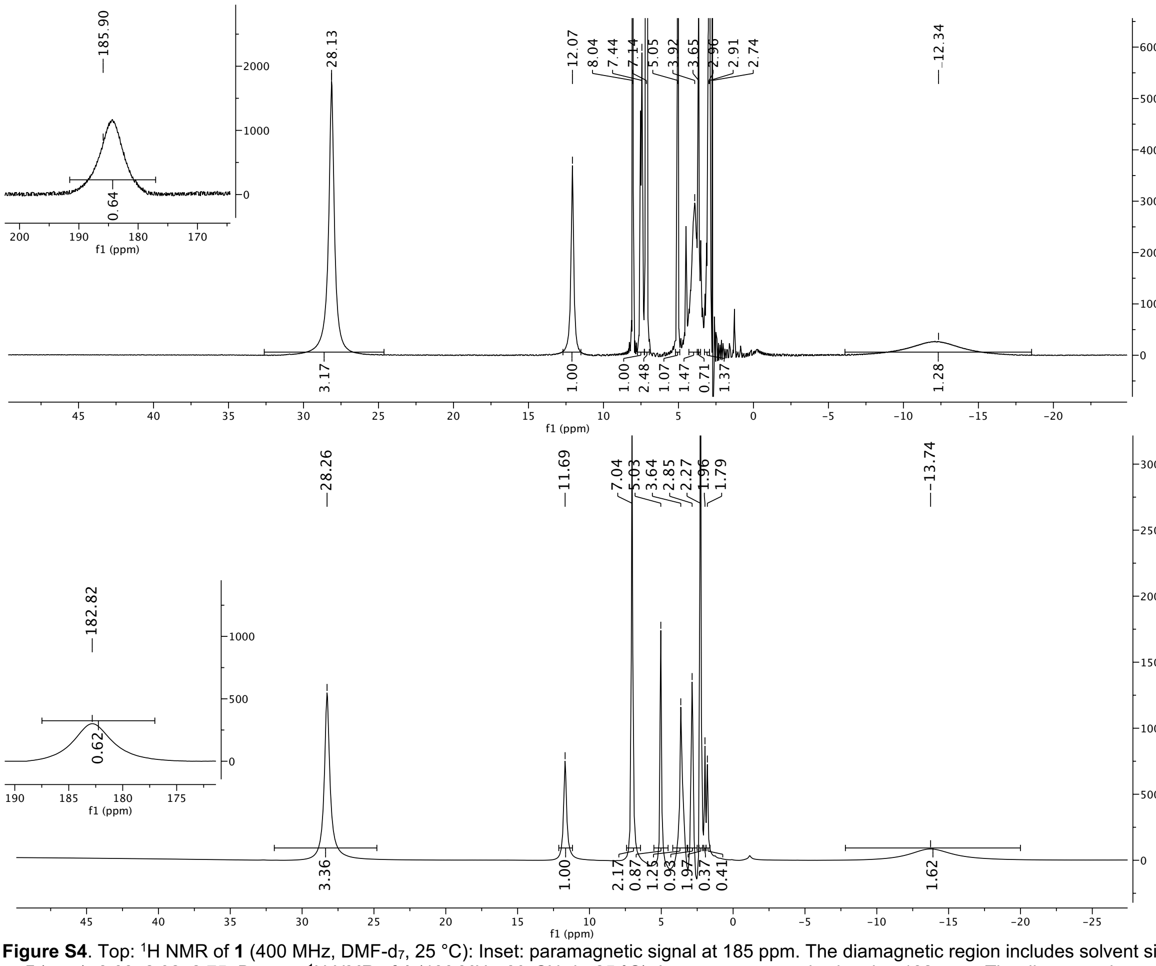

Figure S4. Top: ^{1}H NMR of **1** (400 MHz, DMF-d_7, 25 °C): Inset: paramagnetic signal at 185 ppm. The diamagnetic region includes solvent signals at δ (ppm): 8.03, 2.92, 2.75. Bottom: ^{1}H NMR of **1** (400 MHz, MeCN-d_3, 25 °C): Inset: paramagnetic signal at 182 ppm. The diamagnetic region includes the solvent signal at δ (ppm): 1.94.

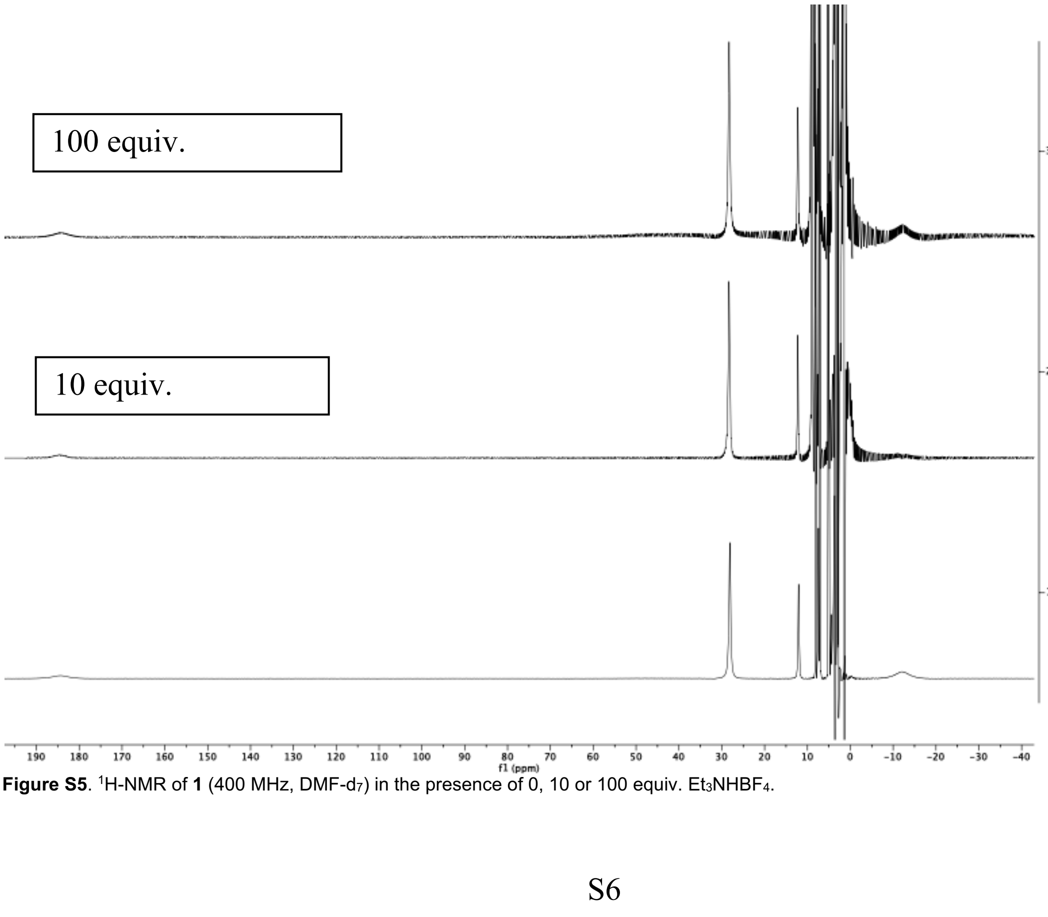

Figure S5. ^{1}H-NMR of **1** (400 MHz, DMF-d_7) in the presence of 0, 10 or 100 equiv. Et_3NHBF_4.

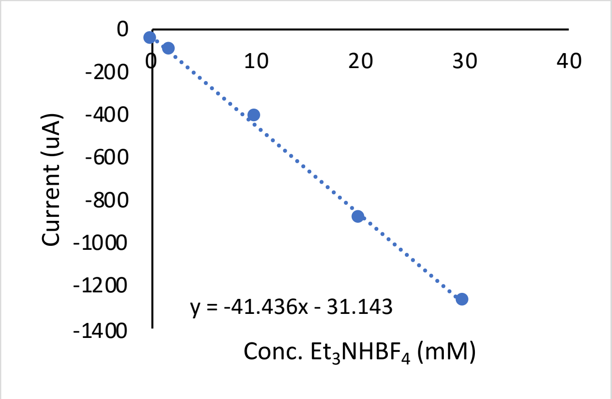

Figure S6. Proportional catalytic peak currents (,$i_{p,c}$) as a function of the acid concentration for **1** (2 mM) in DMF (containing 0.1 M *n*Bu_4NBF_4 as supporting electrolyte)with Et_3NHBF_4 (2, 10, 20, 30 mM).

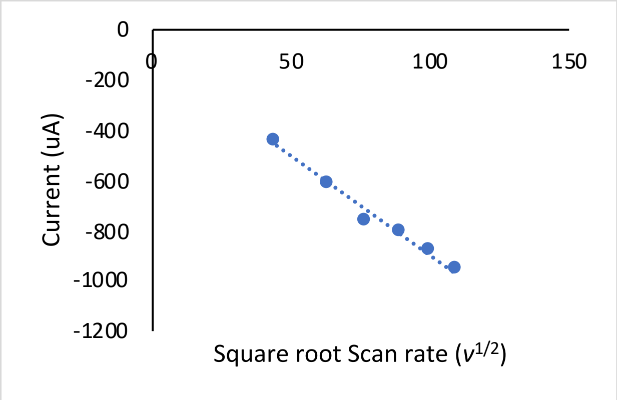

Figure S7. Proportional catalytic peak currents ($i_{p,c}$) as a function of the square root of the scan rate (*v*), indicating a diffusion limited catalytic process for **1** (2 mM) in the presence of 10 mM Et_3NHBF_4 in DMF (containing 0.1 M *n*Bu_4NBF_4 as supporting electrolyte); Scan rates: 1000, 2000, 4000, 6000, 8000, 10000 and 12000 mV.s^{-1}. Working electrode: glassy carbon. Counter electrode: Pt wire. Reference electrode: Ag/Ag(NO_3).

Electrocatalysis Phenol:

Phenol is a weak proton source with a more negative thermodynamic reduction potential ($E°_{HA}$ = <–1.83 V) compared to Et_3NHBF_4 ($E°_{HA}$ = −1.31 V) in DMF.[6] When phenol was added to an electrolyte solution with **1**, a smaller decrease of the oxidative response and a smaller increase in current at the Co(II)/Co(I) couple was observed, resulting in a lower electrocatalytic rate at a lower overpotential (Figure S8). Using equation 3, a k_{obs}(*TOF*) of 8 s^{-1} in presence of 10 mM phenol was determined. The required overpotential is only 130 mV, which is among the lowest overpotentials found for non-aqueous electrocatalytic molecular HER catalysis.

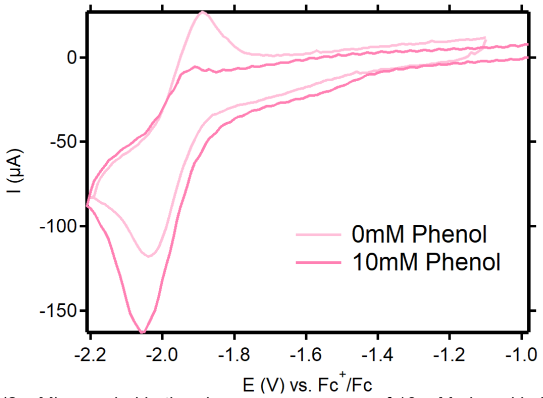

Figure S8. Cyclic voltammogram of **1** (2 mM) recorded in the absence or presence of 10 mM phenol in DMF (0.1 M $n$$Bu_4NBF_4$). Potentials in V vs Fc^+/Fc. Scan rate 2000 mV.s^{-1}. Working electrode: glassy carbon. Counter-electrode: Pt wire. Reference electrode: Ag/Ag(NO_3).

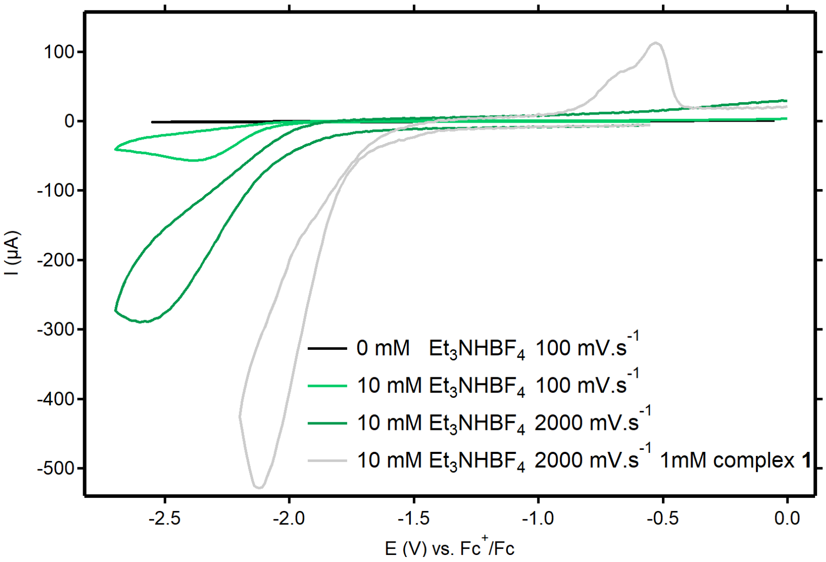

Figure S9. Cyclic voltammograms in the absence (black trace) or in the presence of 10 mM of Et_3NHBF_4 (green traces: scan rate 100 and 2000 mV.s^{-1}) recorded in DMF (containing 0.1 M $n$$Bu_4NBF_4$ as supporting electrolyte). For reference: **1** in the presence of 10 mM Et_3NHBF_4 (gray trace). Working electrode: glassy carbon. Counter-electrode: Pt wire. Reference electrode: Ag/Ag(NO_3). Potentials in V vs Fc^+/Fc.

Electrolysis HER Model-Cell Design

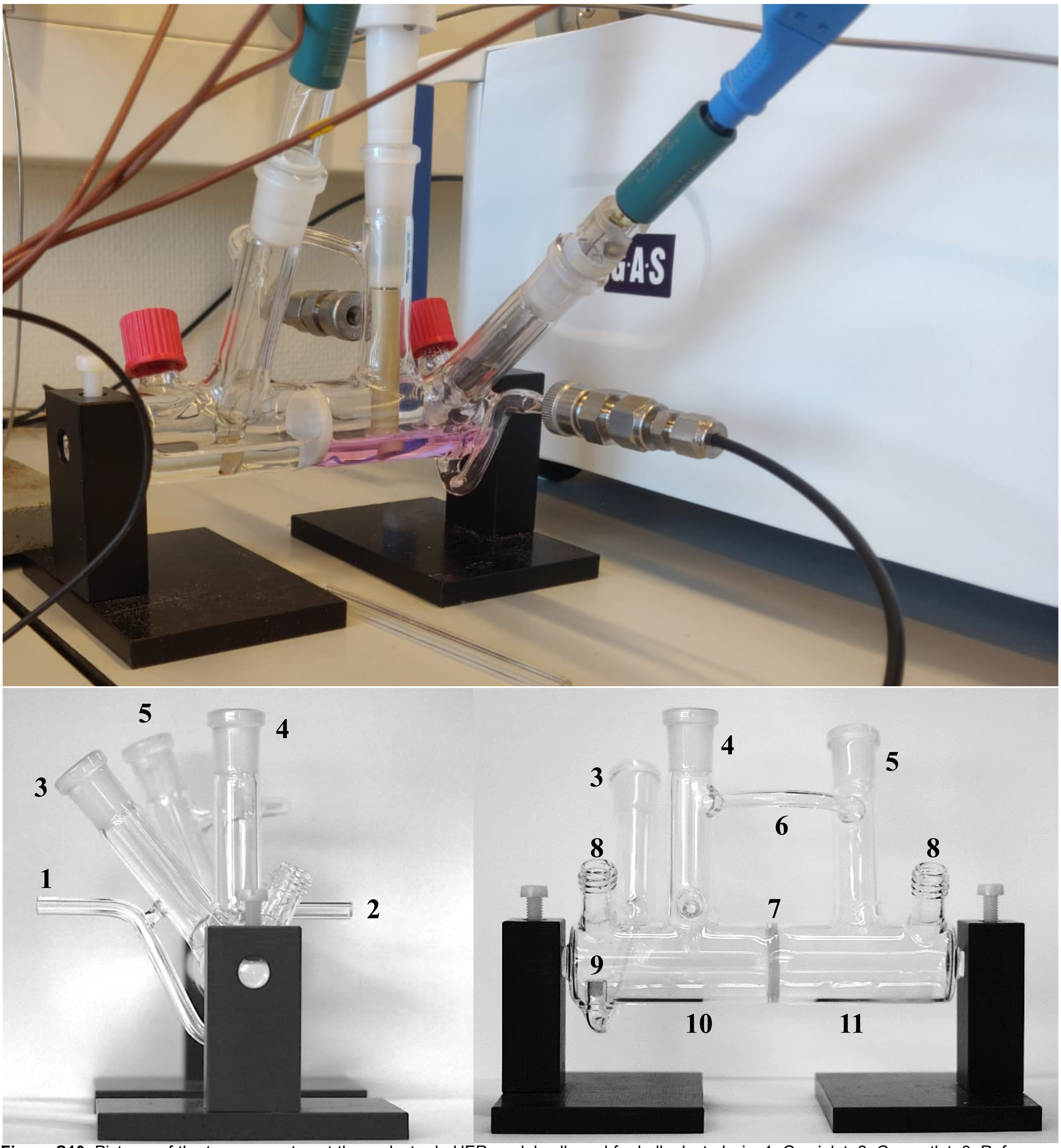

Figure S10. Pictures of the two compartment three-electrode HER model-cell used for bulk electrolysis. 1. Gas inlet, 2. Gas outlet, 3. Reference electrode shaft, 4. Rotating Disk Electrode shaft, 5. Counter electrode shaft, 6. Pressure equalizer tube, 7. Porous P5 glass filter, 8. Opening with screw thread for possible addition or removal of materials, 9. Porous P1 glass filter on gas inlet 10. WE and RE compartment, 11. CE compartment.

Table S1. Stationary (RDE at 0 rpm) bulk electrolysis experiments for H_2 evolution in DMF (0.1 M *n*Bu_4NBF_4) in the presence of 0.1 M Et_3NHBF_4 at -2.00 V vs Fc^+/Fc, coupled to gas chromatography analysis

Catalyst (1mM)	Time (h)	Charge Consumption (C)	Current denisty mA/cm^2	TON[a]	Faradaic efficiency	Overpotential
-	2	0.3	0.18	-	>98%	690 mV
**1**	3	18.7	8.7	9.7	>99%	690 mV

[a]Working electrode: Glassy Carbon (0.196 cm^2). Counter-electrode: Pt plate. Reference electrode: Ag/Ag(NO_3).

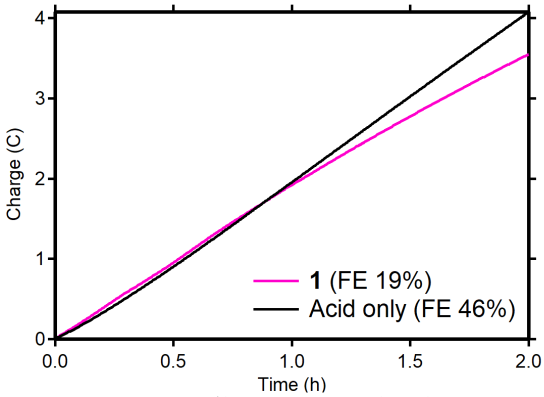

Figure S11. Charge consumed during CPE at −2.00 V versus Fc^+/Fc in the absence (black) and presence (pink) of 1 mM **1**, 0.1 mM AcOH in MeCN (containing 0.1 M *n*Bu_4NBF_4 as the supporting electrolyte). Working electrode: glassy carbon; Counter-electrode: Pt wire; Reference electrode: Ag/Ag(NO_3)

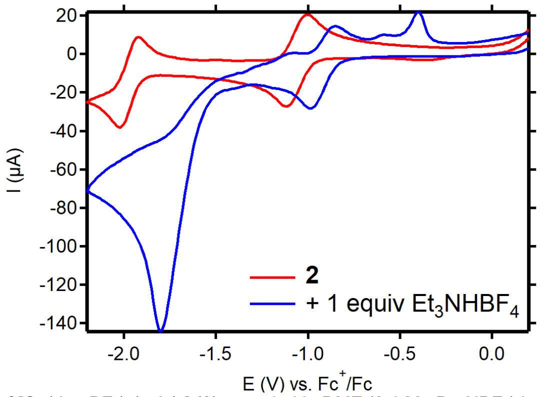

Figure S12.[2] Cyclic voltammograms of [Co(dmgBF$_2$)$_2$(solv)$_2$] (**2**) recorded in DMF (0.1 M *n*Bu$_4$NBF$_4$) in the absence (red trace) or in the presence of 1 equiv. Et$_3$NHBF$_4$ (blue trace). Scan rate 100 mV.s^{-1}. Potentials in V vs Fc$^+$/Fc. Working electrode: glassy carbon. Counter-electrode: Pt wire. Reference electrode: Ag/Ag(NO$_3$).

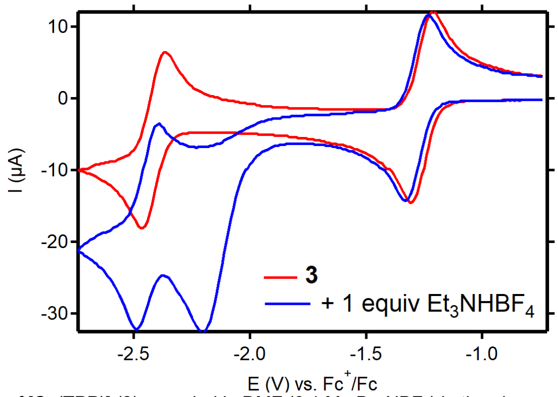

Figure S13.[7] Cyclic voltammograms of [Co(TPP)] (**3**) recorded in DMF (0.1 M *n*Bu$_4$NBF$_4$) in the absence (red trace) or in the presence of 1 equiv. Et$_3$NHBF$_4$ (blue trace). Scan rate 100 mV.s^{-1}. Potentials in V vs Fc$^+$/Fc. Working electrode: glassy carbon. Counter-electrode: Pt wire. Reference electrode: Ag/Ag(NO$_3$).

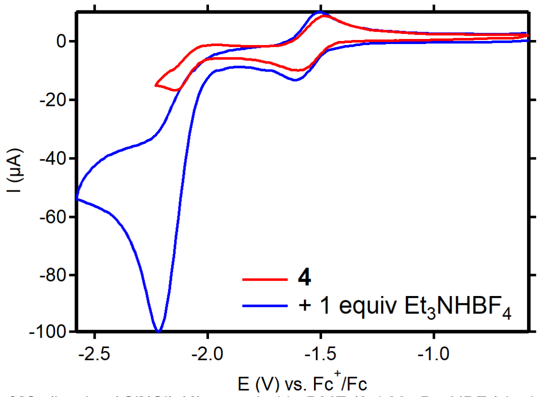

Figure S14.[3] Cyclic voltammograms of [Co(bapbpy)Cl](Cl) (**4**) recorded in DMF (0.1 M *n*Bu$_4$NBF$_4$) in the absence (red trace) or in the presence of 1 equiv. Et$_3$NHBF$_4$ (blue trace). Scan rate 100 mV.s^{-1}. Potentials in V vs Fc$^+$/Fc. Working electrode: glassy carbon. Counter-electrode: Pt wire. Reference electrode: Ag/Ag(NO$_3$).

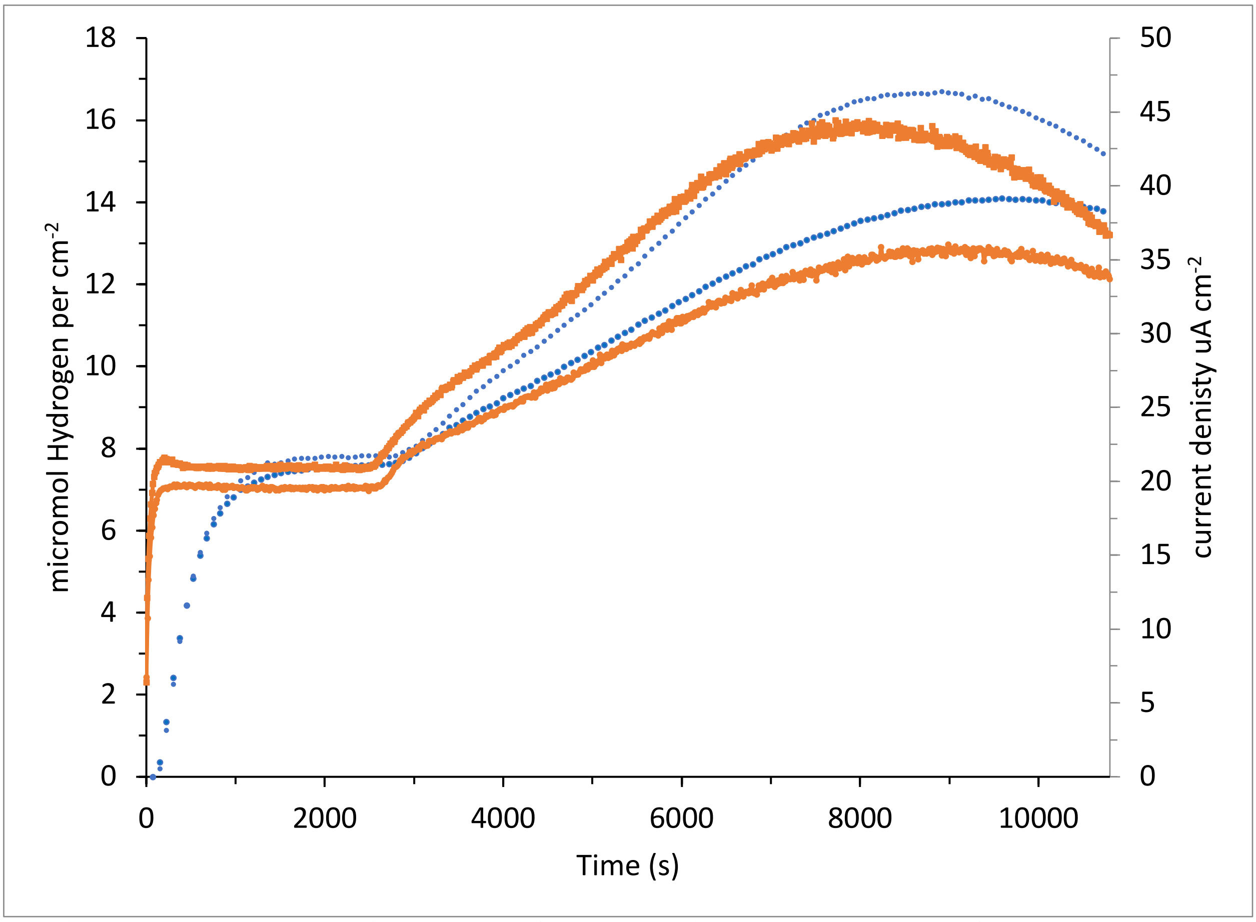

Figure S15. Controlled potential electrolysis (duplo measurement) in the presence of 1 mM **4**, 0.1 M Et_3NHBF_4 in DMF (containing 0.1 M $n$$Bu_4NBF_4$ as the supporting electrolyte) at –2.00 V vs Fc^+/Fc at 2000 rpm. Blue dotted traces (left vertical axis): Hydrogen production. Orange (right vertical axis): current density. Indicating the reproducibility of the suggested structural changes after 45 minutes (2700 second).

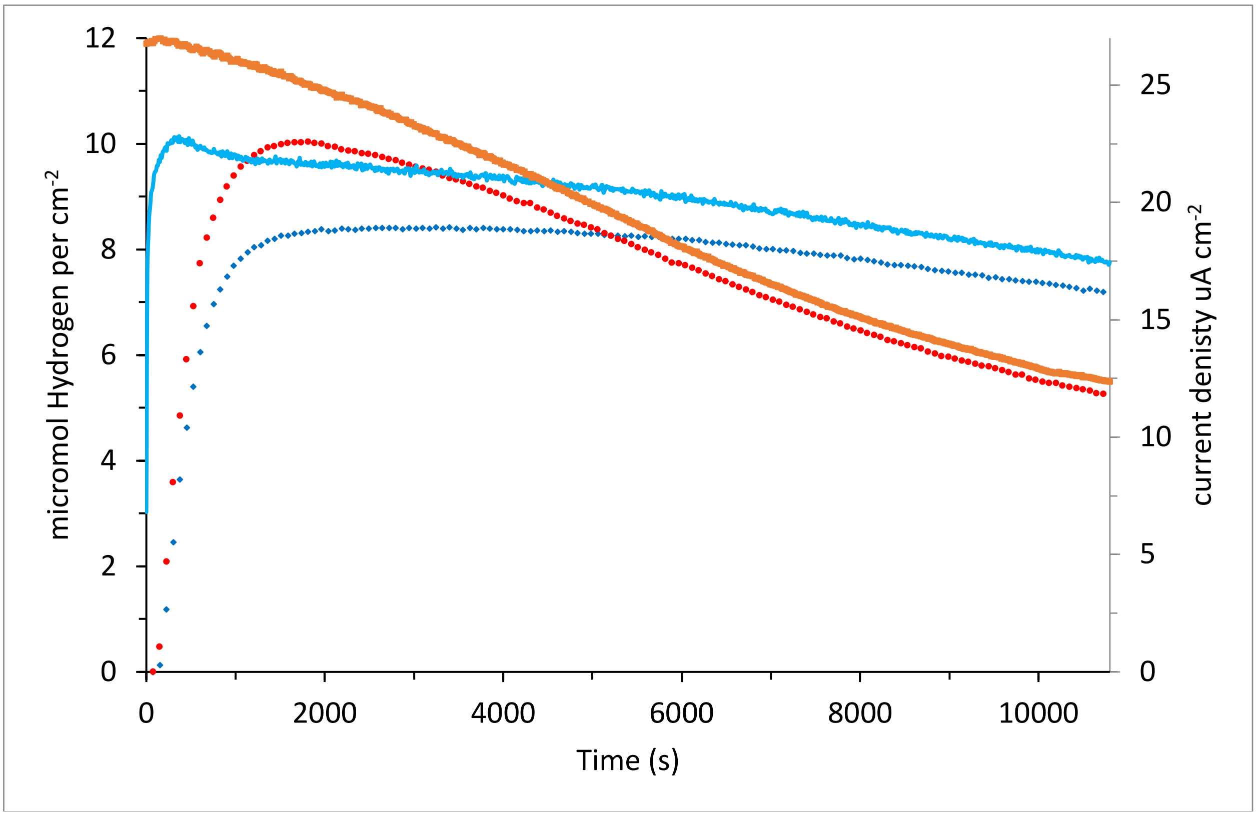

Figure S16. Data corresponding to Figure 8, pink traces: raw data showing that the deposition on the electrode surface is an active catalytic material for HER. Left axis (hydrogen production) blue dots: initial CPE (pristine electrode), red dots: rinse test CPE (modified electrode).
Right axis (current density) Light cyan trace: initial CPE (pristine electrode), Orange trace: rinse test (modified electrode)
Solution: 0.1 mM Et_3NHBF_4 (containing 0.1 M $n$$Bu_4NBF_4$ as the supporting electrolyte) at –2.00 V vs Fc^+/Fc, 2000 rpm. Initial: + 0.5 mM **1**, rinse test: fresh solution with no complex added.

Note: The higher initial current for the rinse test is caused by the direct presence of the active deposition in the vicinity of a fresh acid solution.

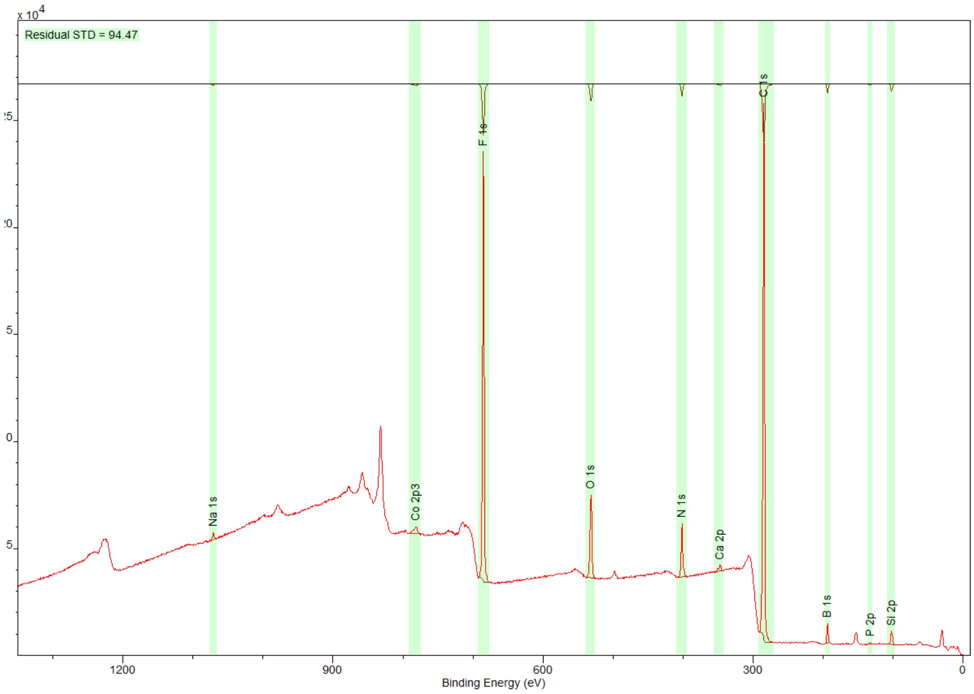

Relative percentage of selected atoms

C	68.5%
N	4.04%
O	4.29%
B	5.0%
F	15.7%
Co	0.23%

Figure S17. XPS spectrum of the electrode deposit after 3 h electrolysis in DMF 0.1 M Et_3NHBF_4,0.1 M $n$$Bu_4NBF_4$, 1 mM **1** at –2.00 V vs. Fc^+/Fc and its relative atomic ratios. High percentages of carbon are caused using carbon tape during the analysis.

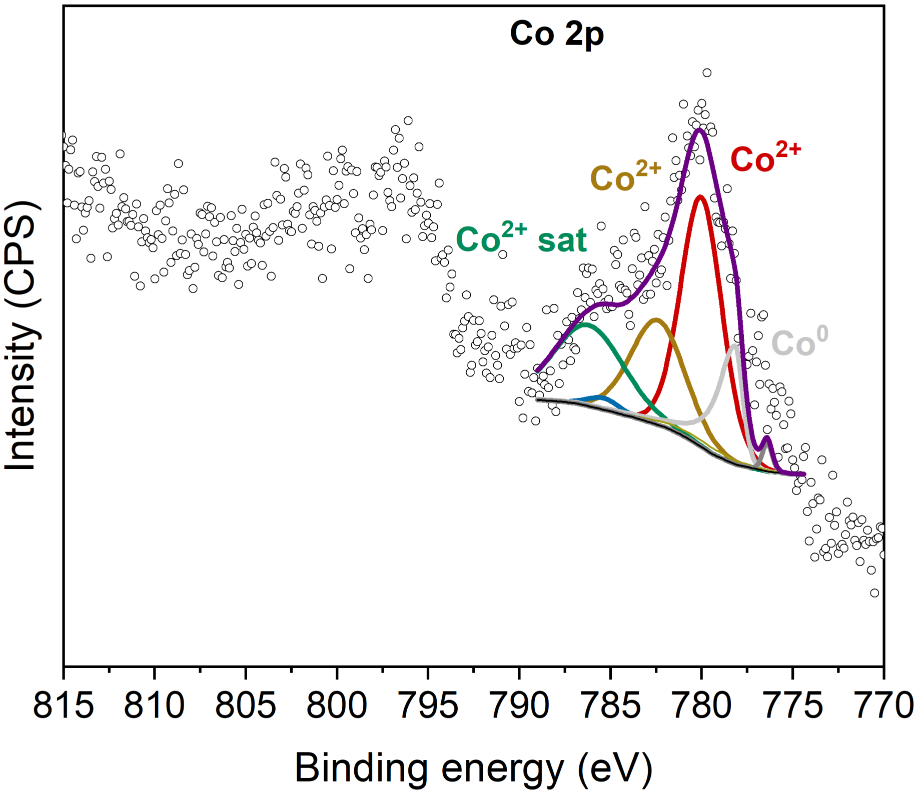

Figure S18. XPS spectrum of the Co region from the electrode deposit after 3 h electrolysis in DMF 0.1 M Et_3NHBF_4,0.1 M $n$$Bu_4NBF_4$, 1 mM **1** at –2.00 V vs. Fc^+/Fc and its relative atomic ratios. The satellite peaks indicate a large contribution of the 2^+ oxidation state. The spectrum was fitted according to the work of Biesinger et. al.[8]

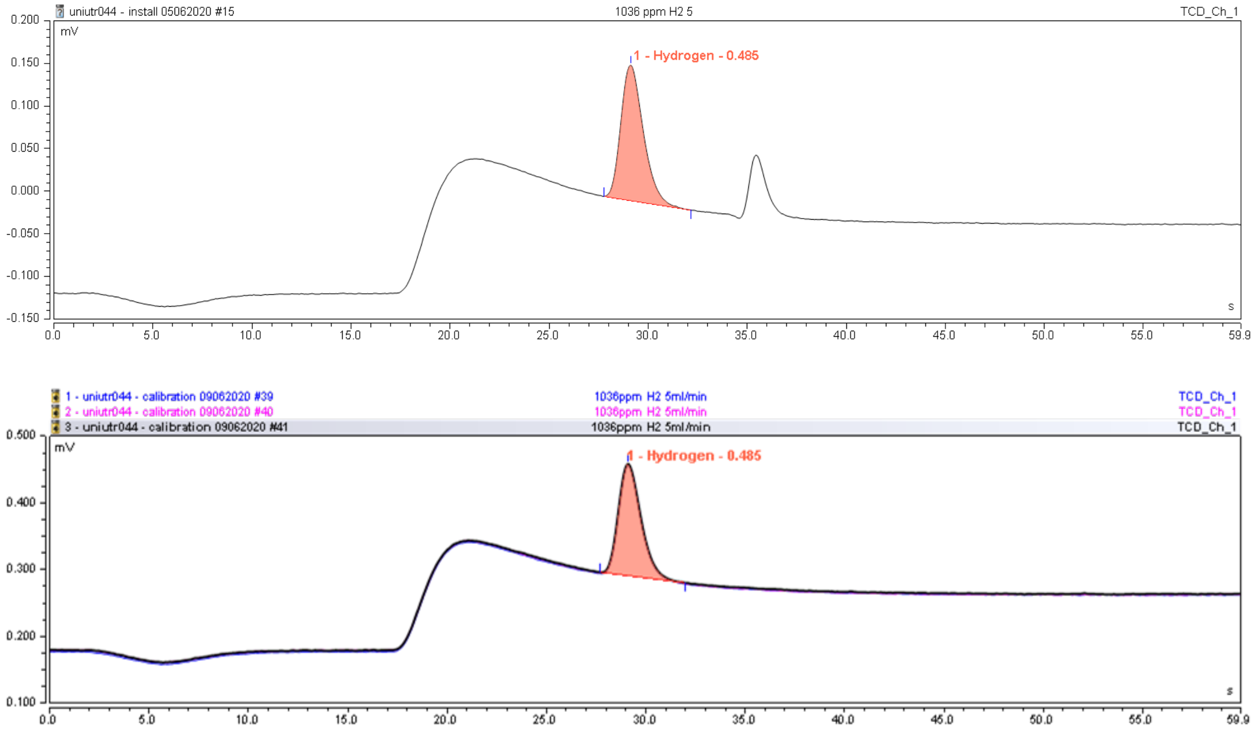

Figure S19. GC-TCD chromatograms of a 1036 ppm H_2 in N_2 mix gas overtime. Top: t = 60 s hydrogen at 29.0 retention time and residual oxygen at 35.4 retention time. Bottom: t = 600 s no oxygen is observed.

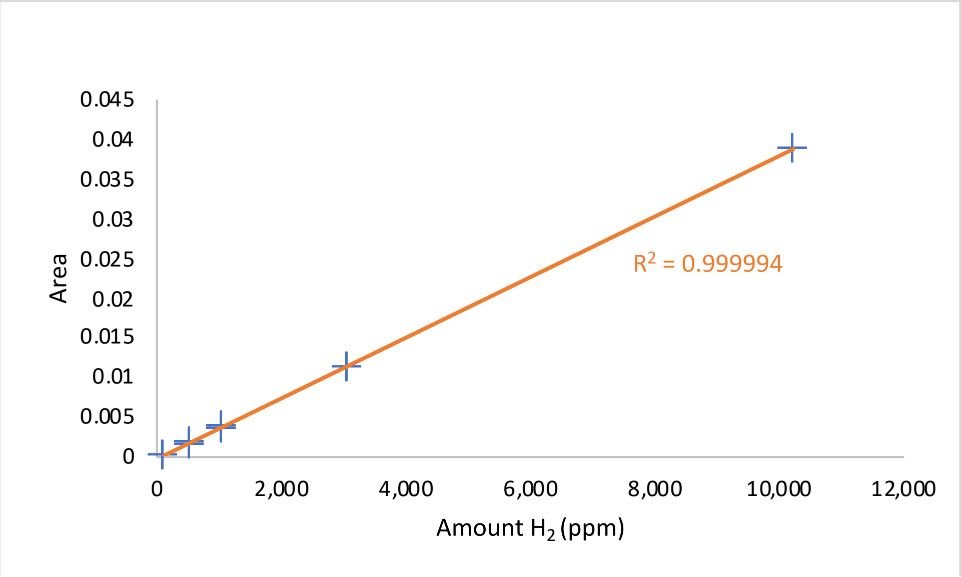

Figure S20. GC-TCD calibration with five H_2/N_2 mixtures of known composition containing 97.5, 508, 1036, 3046, 10210 ppm H_2 gas. Number of calibration points: 45.

[1] P. Ghosh, S. de Vos, M. Lutz, F. Gloaguen, P. Schollhammer, M.-E. Moret, R. J. M. Klein Gebbink, *Chemistry – A European Journal* **2020**, *26*, 12560-12569.
[2] X. Hu, B. M. Cossairt, B. S. Brunschwig, N. S. Lewis, J. C. Peters, *Chemical Communications* **2005**, 4723-4725.
[3] N. Queyriaux, D. Sun, J. Fize, J. Pécaut, M. J. Field, M. Chavarot-Kerlidou, V. Artero, *Journal of the American Chemical Society* **2020**, *142*, 274-282.
[4] K. Izutsu, P. International Union of, C. Applied, C. Commission on Electroanalytical, *Acid-base dissociation constants in dipolar aprotic solvents*, Blackwell Scientific Publications, Oxford, **1990**.
[5] U. J. Kilgore, J. A. S. Roberts, D. H. Pool, A. M. Appel, M. P. Stewart, M. R. DuBois, W. G. Dougherty, W. S. Kassel, R. M. Bullock, D. L. DuBois, *Journal of the American Chemical Society* **2011**, *133*, 5861-5872.
[6] G. A. N. Felton, R. S. Glass, D. L. Lichtenberger, D. H. Evans, *Inorganic Chemistry* **2006**, *45*, 9181-9184.
[7] B. B. Beyene, S. B. Mane, C.-H. Hung, *Journal of The Electrochemical Society* **2018**, *165*, H481-H487.
[8] M. C. Biesinger, B. P. Payne, A. P. Grosvenor, L. W. M. Lau, A. R. Gerson, R. S. C. Smart, *Applied Surface Science* **2011**, *257*, 2717-2730.
